# Small Ruminant Nor98 Prions Share Biochemical Features with Human Gerstmann-Sträussler-Scheinker Disease and Variably Protease-Sensitive Prionopathy

**DOI:** 10.1371/journal.pone.0066405

**Published:** 2013-06-24

**Authors:** Laura Pirisinu, Romolo Nonno, Elena Esposito, Sylvie L. Benestad, Pierluigi Gambetti, Umberto Agrimi, Wen-Quan Zou

**Affiliations:** 1 Department of Veterinary Public Health and Food Safety, Istituto Superiore di Sanità, Rome, Italy; 2 Norwegian Veterinary Institute, Oslo, Norway; 3 Department of Pathology, National Prion Disease Pathology Surveillance Center, Case Western Reserve University School of Medicine, Cleveland, Ohio, United States of America; The Scripps Research Institute Scripps Florida, United States of America

## Abstract

Prion diseases are classically characterized by the accumulation of pathological prion protein (PrP^Sc^) with the protease resistant C-terminal fragment (PrP^res^) of 27–30 kDa. However, in both humans and animals, prion diseases with atypical biochemical features, characterized by PK-resistant PrP internal fragments (PrP^res^) cleaved at both the N and C termini, have been described. In this study we performed a detailed comparison of the biochemical features of PrP^Sc^ from atypical prion diseases including human Gerstmann-Sträussler-Scheinker disease (GSS) and variably protease-sensitive prionopathy (VPSPr) and in small ruminant Nor98 or atypical scrapie. The kinetics of PrP^res^ production and its cleavage sites after PK digestion were analyzed, along with the PrP^Sc^ conformational stability, using a new method able to characterize both protease-resistant and protease-sensitive PrP^Sc^ components. All these PrP^Sc^ types shared common and distinctive biochemical features compared to PrP^Sc^ from classical prion diseases such as sporadic Creutzfeldt-Jakob disease and scrapie. Notwithstanding, distinct biochemical signatures based on PrP^res^ cleavage sites and PrP^Sc^ conformational stability were identified in GSS A117V, GSS F198S, GSS P102L and VPSPr, which allowed their specific identification. Importantly, the biochemical properties of PrP^Sc^ from Nor98 and GSS P102L largely overlapped, but were distinct from the other human prions investigated. Finally, our study paves the way towards more refined comparative approaches to the characterization of prions at the animal–human interface.

## Introduction

Transmissible spongiform encephalopathies (TSEs), or prion diseases, are fatal and transmissible neurodegenerative disorders that occur in human and animals. They comprise a broad spectrum of clinico-pathological variants, which have been found to be associated with distinct prion strains. This is the case of sporadic Creutzfeldt-Jakob disease (sCJD) sub-types in human [1]–[5], classical scrapie strains in small ruminants [6], [7], and the different bovine spongiform encephalopathy (BSE) types in cattle, namely BSE-C, BSE-L and BSE-H [8]–[12]. The prion hypothesis postulates that prions would be composed mainly or exclusively of PrP^Sc^, a misfolded form of the cellular prion protein (PrP^C^), forming highly-ordered aggregates insoluble in detergents and partially resistant to proteolysis [13]. The prion hypothesis equates prion strains to different self-propagating conformational variants of PrP^Sc^, mirrored by the diversity of physicochemical properties of PrP^Sc^ observed in human and animal prion diseases [14]–[20]. Typically, proteinase K (PK) treatment hydrolyses the N-terminus of PrP^Sc^, resulting in partially PK-resistant C-terminal PrP fragments (PrP^res^), also designated PrP^27–30^ [13]. Currently, the electrophoretic mobility and glycoform ratio of PrP^27–30^ are the basis for the biochemical classification of TSEs, although the biological identification of prion strains is still based on biological strain typing in rodents.

Pioneering studies in Gerstmann-Sträussler-Scheinker disease (GSS), familial human prion diseases associated with different PrP mutations, showed that purified amyloid preparations and PrP^res^ aggregates obtained by *in vitro* proteolysis contained atypical 7–8 kDa PrP fragments, with ragged N and C termini [21]–[27], which were mainly composed of mutant PrP alleles [21], [25], [28]–[30]. Recently, however, PrP^res^ features reminiscent of GSS were found in a newly described human sporadic prion disorder, variably protease sensitive prionopathy (VPSPr) [31], [32]. In VPSPr both PK -sensitive and -resistant PrP^Sc^ species were characterized, with the most abundant PK resistant fragment being a ∼6–7 kDa PrP^res^ similar to GSS [31], [32]. Interestingly, VPSPr was not associated with mutations in the PrP ORF, showing that the low molecular weight (MW) PrP^res^ may derive from wild type human PrP, and raising the question of whether VPSPr might represent the sporadic form of GSS [33]. A new study also demonstrated that VPSPr shared PrP^Sc^ features with a known familial CJD linked to a valine to isoleucine mutation at residue 180 of PrP (fCJDV180I), exhibiting similar patterns of glycosylation, protease cleavage, and immunoreactivity preference [34]. Interestingly, the same group also identified a unique glycoform-selective prion formation pathway in the two diseases [34].

A prion disease with a similar low MW PrP^res^, Nor98 or atypical scrapie, has been also described in small ruminants [35]. Firstly recognised in Norwegian sheep in 1998 [36], a retrospective study in UK back-dated the presence of Nor98 cases to at least 1989, suggesting that these cases existed in small ruminant populations for years without being detected [37]. Since 2002, Nor98 has been identified in most of EU Member States [35], Canada [38], USA [39] and New Zealand [40]. Unlike classical scrapie, Nor98 occurs with a sporadic distribution [41], [42] and is diagnosed mainly in aged sheep and goats with specific PrP polymorphisms [43], [44]. Although Nor98 is supposed to be a spontaneous disorder [35], [42], [45], it is diagnosed at a relatively high frequency in the EU, with a prevalence of ∼4 over 10,000 examined [46]. Despite the lack of epidemiological evidence for natural transmission of Nor98, its transmissibility has been demonstrated in both ovinised transgenic mice [47] and sheep [48], [49], and infectivity was also detected in peripheral tissues [49], [50], indicating that potentially infectious material might enter the food chain.

In this study we aimed at comparing the PrP^Sc^ properties of Nor98, GSS and VPSPr cases, getting insight into their apparent similarity and investigating possible relationships among them, particularly at the animal-human interface. To this aim we set up refined biochemical techniques for inter-species comparative epitope mapping of PrP^res^ fragments and for determining the conformational stability of both PK sensitive and PK resistant PrP^Sc^ species. Our findings show that GSS, VPSPr and Nor98 share common and distinctive biochemical features, supporting the notion that these atypical forms of PrP^Sc^ are not uncommon and characterize a group of prion diseases with different origins (genetic and spontaneous forms) occurring in different hosts. Within this group, we found PrP^Sc^ biochemical signatures specific to VPSPr, GSS A117V and GSS F198S, while the phenotypic profile of GSS P102L largely overlapped with that of Nor98.

## Results

### GSS-like PrP^res^ Profile in Nor98

Previous studies reported partially discrepant observations of the Nor98 PrP^res^ electrophoretic profile [36], [37], [51]–[57], probably due to the different antibodies and protocols for PK digestion used [35]. The most distinguishing biochemical feature of Nor98 was a low MW PrP^res^ fragment [36], although studies which used a mild PK protocol also described variable amounts of higher MW, partially glycosylated PrP^res^ fragments [52], [53], [57].

**Figure 1:**
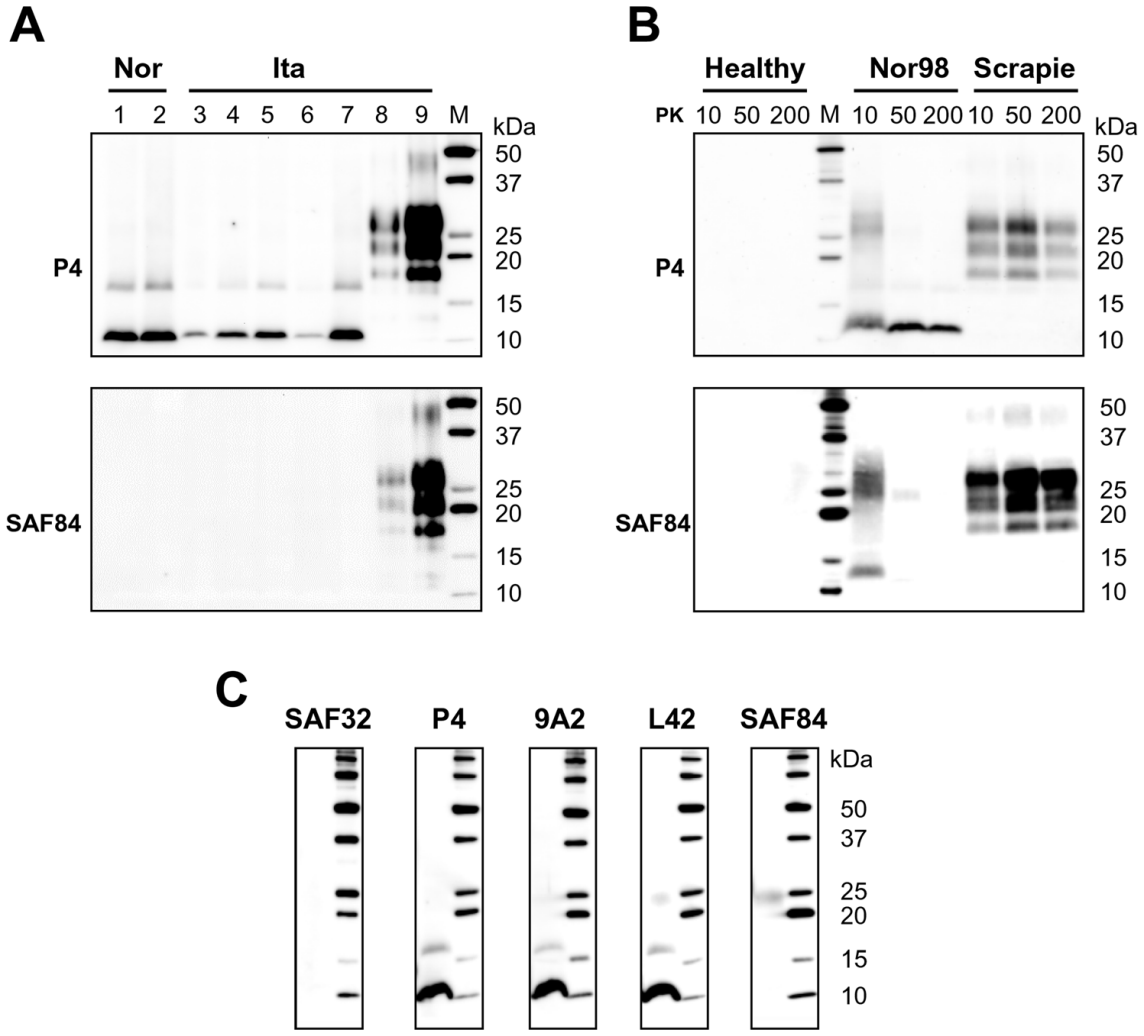
Western blot analysis of Nor98 isolates. **A:** Representative western blots of PrP^res^ from Nor98 isolates (Table S1). Lanes 1–2, Norwegian sheep Nor98 (AFRQ/AFRQ); lanes 3–5, Italian goat Nor98 (ALRQ/ALHQ); lanes 6–7, Italian sheep Nor98 (AFRQ/AFRQ and ALRR/ALHQ, respectively); lane 8, Italian goat scrapie (ALRQ/ALRQ); lane 9, Italian sheep scrapie (ALRQ/AFRQ). All samples were digested at 200 µg/ml PK. Membranes were probed with P4 (top) and SAF84 (bottom). **B:** Effect of PK digestion on Nor98 PrP^Sc^. WB analysis of healthy, Nor98 (Italian sheep, AFRQ/AFRQ) and scrapie (Italian sheep, ALRQ/ALRQ) sheep brain homogenate treated with 10, 50 and 200 µg/ml PK. Replica blots were probed with P4 (top) and SAF84 (bottom). MW marker was loaded as indicated (M). **C:** Epitope mapping of Nor98 (Norwegian sheep AFRQ/AFRQ) PrP^res^ produced after 200 µg/ml PK treatment. Membranes were probed with different mAbs, as indicated on the top of each blot. MW markers were loaded into the last lane of each blot. **A-C:** MW marker was loaded as indicated (M).

**Figure 2:**
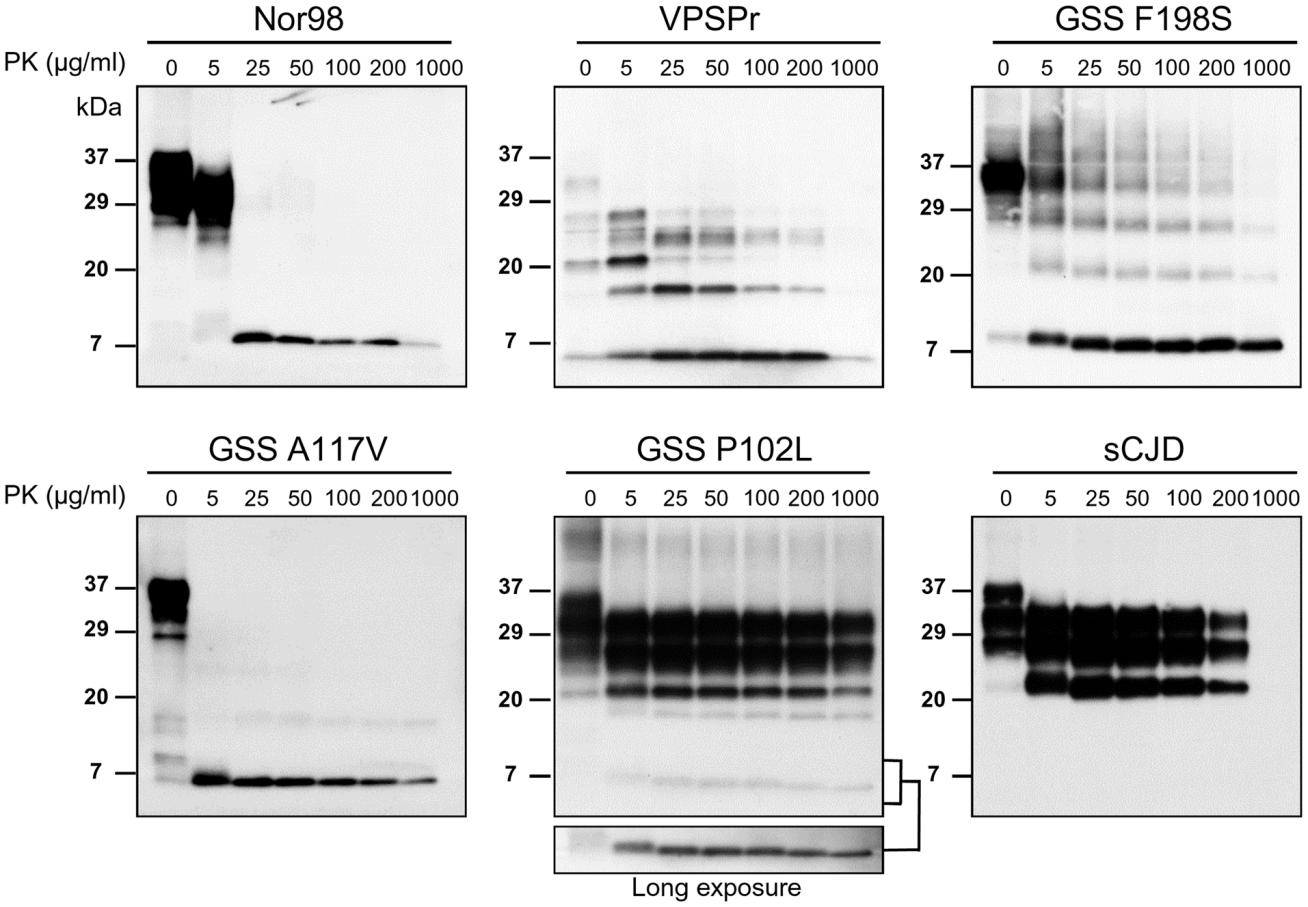
Titration of proteinase K digestion. Representative western blots of brain homogenates from Nor98 (#2), VPSPr (#7), GSS F198S (#11), A117V (#13), P102L (#15) and sCJD treated with increasing concentrations of PK (0–1000 µg/ml). Given the various PK-cleavage sites of the PrP^Sc^ types under investigation which affects mAbs binding to PrP^res^ (see Fig. 3), membranes stained with different antibodies were selected in order to show the PK resistance of strong PrP^res^ in each sample. Membranes were probed with 12B2 for Nor98 and GSS P102L, 9A2 for GSS A117V, 1E4 for VPSPr, and 3F4 for GSS F198S and sCJD samples.

Sheep (n = 63) and goat (n = 14) Nor98 isolates identified in Italy to date [43], [56], [58] showed a uniform WB pattern when analysed by the ISS discriminatory western blot, which is based on strong PK treatment and parallel testing with mAbs SAF84 and P4 [56], [59], [60]. By direct comparison, Italian and Norwegian Nor98 isolates shared the same PrP^res^ type regardless of the host species and the PrP genotype (Fig. 1A and Table S1). Indeed, after treatment with 200 µg/ml PK, a major ∼11 kDa PrP^res^ fragment, and a minor ∼16 kDa PrP^res^ fragment were detected in all isolates by P4, but not by SAF84 (Fig. 1A). These distinctive low MW PrP^res^ fragments produced after 200 µg/ml PK were highly reproducible among samples and both fragments were recognised after immunodetection with P4, 12B2, 9A2 and L42, but not with mAbs N-terminal to P4 or C-terminal to L42 (Fig. 1C). As previously suggested by others [54] the minor PrP^res^ at ∼16 kDa might be interpreted as a dimer of the low MW fragment, as they shared the same antibody reactivity. Notably, the experiments reported in the following sections, aimed at comparing Nor98 with human samples, were performed with 15% acrylamide gels (see materials and methods) and showed that the apparent MW of the main PrP^res^ fragment was ∼7 kDa (Fig. 2), in line with epitope mapping experiments (see below).

To look for mild protease resistant PrP fragments (mild PrP^res^) we studied the kinetics of PrP^Sc^ degradation by PK in Nor98, classical scrapie and healthy brains (Fig. 1B). Classical scrapie was characterized by the typical PrP^27–30^ at all PK concentrations, i.e. three bands corresponding to N-terminally truncated diglycosylated, monoglycosylated and non-glycosylated PrP^res^. In contrast, Nor98 was characterized by PrP^res^ fragments with different degree of resistance to PK. Indeed, at 10 µg/ml PK, both SAF84 and P4 bound poorly defined PrP smears of high MW (apparent MW ∼25–33 kDa) and low MW (apparent MW ∼13–14 kDa), while at 50–200 µg/ml PK only P4 detected the low MW PrP^res^ described above (strong PrP^res^). Experiments performed with a broader range of PK concentrations (Fig. S1) confirmed that in Nor98, but not in classical scrapie, it was possible to distinguish mild and strong PrP^res^ fragments, with high MW PrP^res^ being relatively sensitive to PK (PK_1/2_ between 10 to 30 µg/ml) compared to the ∼10 times stronger PK resistance of low MW PrP^res^ and of PrP^27–30^ in classical scrapie (Fig. S1). Similar findings have been reported in GSS A117V [23] and VPSPr [32].

Overall, all Nor98 isolates contained highly PK resistant PrP^res^ aggregates, with the main PrP^res^ being a non-glycosylated internal fragment, cleaved at both the N and C termini, which represent the distinctive biochemical feature of Nor98. This biochemical signature, unique among animal TSEs, is reminiscent of PrP^res^ observed in human prion disorders such as GSS and VPSPr.

In order to compare the conformation of PrP^Sc^ aggregates in Nor98, VPSPr and GSS we selected 2 Nor98 cases, 6 GSS cases, 2 each with A117V, F198S and P102L PrP mutations, and 8 VPSPr cases, either 129 MM, 129 MV or 129 VV at PrP codon 129 (Table 1). In GSS P102L cases, two distinct biochemical phenotypes have been reported [21], [22], with PrP^res^ accumulating either as PrP^27–30^ accompanied by the 7–8 kDa PrP^res^ or exclusively as 7–8 kDa PrP^res^. One of the cases included in our selection (ID #16) had 7–8 kDa PrP^res^ without PrP^27–30^, while the other (ID #15) showed 7–8 kDa PrP^res^ in the cerebellum, but PrP^27–30^ was also evident in other brain areas (Fig. S2).

**Table 1:** Human and ovine cases used for comparative analyses.

Disease	ID	Genotype^a^	Brain Area^b^	[GdnHCl]_1/2_ c
Nor98	#1	AFRQ/AFRQ	Cer	1.48±0.04
	#2	ALRR/ALHQ	Cer	1.44±0.01
VPSPr	#3	V129V	Fr cx	2.18±0.04
	#4	V129V	Fr cx	2.00±0.04
	#5	V129V	Fr cx	2.36±0.08
	#6	M129V	Fr cx	1,55±0,22
	#7	M129V	Fr cx	1,85±0,14
	#8	M129V	Fr cx	1,50±0,05
	#9	M129M	Fr cx	1,69±0,06
	#10	M129M	Fr cx	1,65±0,04
GSS	#11	F198S-V129V	Fr cx	2.89±0.14
	#12	F198S-M129V	Fr cx	2.91±0.09
	#13	A117V-M129V	Fr cx	1.61±0.03
	#14	A117V-V129V	Fr cx	1.36±0.04
	#15	P102L-M129M	Fr cx	2.41±0.09
			Cer	2.77±0.08
	#16	P102L-M129V	Fr cx	1.53±0.02
			Cer	1.63±0.16

a Ovine polymorphism: amino acids at codon 136, 141, 154 and 171 of ovine PrP gene; human mutation at codon 102, 117 or 198 and polymorphism at codon 129 of human PrP gene.

b Cer: cerebellum; Fr cx: frontal cortex.

c Mean ± SEM.

### PK Titration of PrP^Sc^ from Nor98, GSS and VPSPr

**Figure 3:**
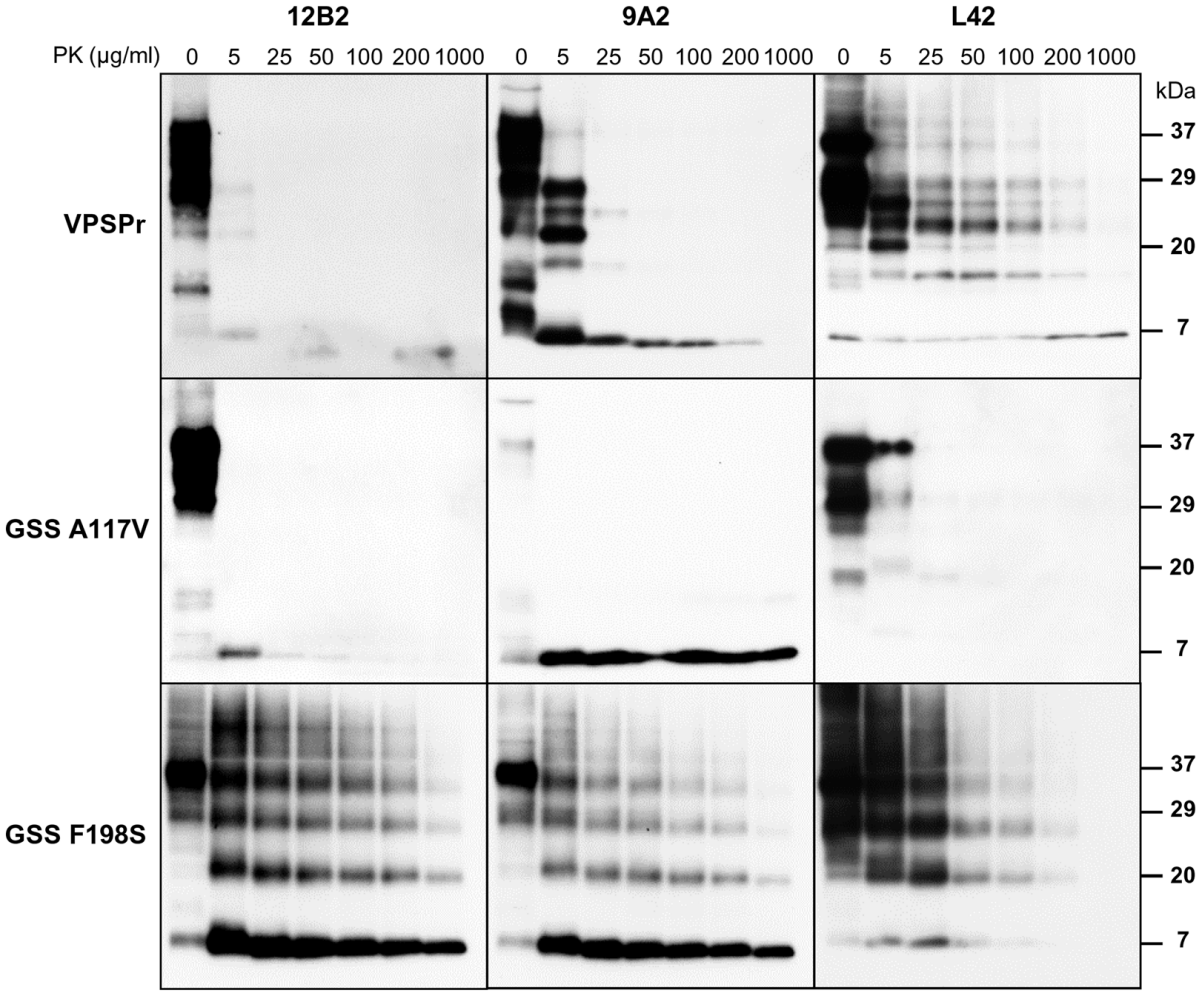
Epitope mapping of PrP^Sc^ treated with different PK concentrations. PK digestion curves of VPSPr (#7), GSS A117V (#13) and GSS F198S (#11) were probed with 12B2, 9A2 and L42.

Given that PK concentration was crucial to faithfully characterize Nor98 PrP^res^ fragments, at first we co-analysed sheep and human samples by PK titration. These experiments confirmed that mild and strong PrP^res^ fragments were also detectable in several human samples (Fig. 2). As previously reported [32], in VPSPr two mild PrP^res^ fragments, of ∼20 and ∼26 kDa, were digested at >5 µg/ml PK (Fig. 2), similar to mild PrP^res^ in Nor98 (Fig. 1B and Fig. 2). Three other VPSPr PrP^res^ fragments, including the most abundant one at ∼6–7 kDa, were strongly resistant to proteolysis (Fig. 2). In GSS cases, evidence for mild PrP^res^ was less consistent, with case to case variations (Fig. 2 and Fig. 3). In contrast, strong PrP^res^ migrating variably at 6–8 kDa was detected in all samples, accompanied by ladder-like, higher MW strong PrP^res^ fragments (Fig. 2).

Overall, all samples contained strongly PK resistant low MW PrP^res^ in the range of 6–8 kDa that were still detectable at 1 mg/ml PK. The degree of PK resistance of PrP^res^ aggregates was similar in all samples and also comparable with that observed in classical sCJD (Fig. 2) and scrapie (Fig. S1). Evidence for a similar PK resistance of classical and atypical PrP^Sc^ aggregates was also obtained by analysing GSS P102L with both PrP^27–30^ and 8 kDa PrP^res^, in which the two molecular components showed closely similar degrees of PK resistance (Fig. 2).

By comparing the dynamics of N- and C-terminal PK cleavage at flanking epitopes with 12B2, 9A2 and L42, we found that sequential trimming of the N- and C-termini was heterogeneous among samples. Indeed, the 12B2 epitope was sequentially cleaved between 5 and 100 µg/ml PK in GSS A117V and VPSPr samples, but not in GSS F198S (Fig. 3). Conversely, the C-terminal L42 epitope was sensitive to PK in GSS A117V, partially sensitive in GSS F198S, and resistant in VPSPr (Fig. 3). These experiments suggest that the PK cleavage sites of PrP^res^ aggregates in GSS and VPSPr may vary at both N- and C-termini and that the ability to detect epitopes near the cleavage sites strongly depends on the PK concentration used.

### Comparison of PrP^res^ Internal Fragments from Nor98, GSS and VPSPr by Epitope Mapping

Based on PK titration experiments we thus selected PK at 100 µg/ml as the most convenient dose to compare by epitope mapping low MW PrP^res^ species from all samples reported in Table 1. To this aim, 9 mAbs with epitopes spanning from the octarepeat to residue 160 (Table 2), were selected because i) they bound ovine and human PrP molecules equally well and ii) they did not show disease specific conformational preferences.

**Table 2:** Monoclonal antibodies used for epitope mapping.

mAbs	Epitope^a^	VPSPr	A117V	F198S	P102L	Nor98
SAF32	octarepeat^b^	−	−	+	+	−
12B2	89–93	−	+/−	+	+	+
9A2	99–101	+/−	+	+	+	+
8G8	97–104	+	+	+	+	+
6D11	94–110	+	+	+	+	+
F89	139–142	+	+	+	+	+
L42	145–150	+	−	+/−	+	+
12F10	144–152	+	−	−	−	−
SAF60	157–161	−	−	−	−	−

a Epitope on human PrP.

b Repeat region aa 59–65, 67–73, 75–81, 83–89. Epitope reference: SAF32, 8G8,12F10, SAF60 [61]; 12B2 [62]; 9A2 [63]; 6D11 [64], [65]; F89 [66]; L42 [54].

−no signal; +/−weak signal; +strong signal.

**Figure 4:**
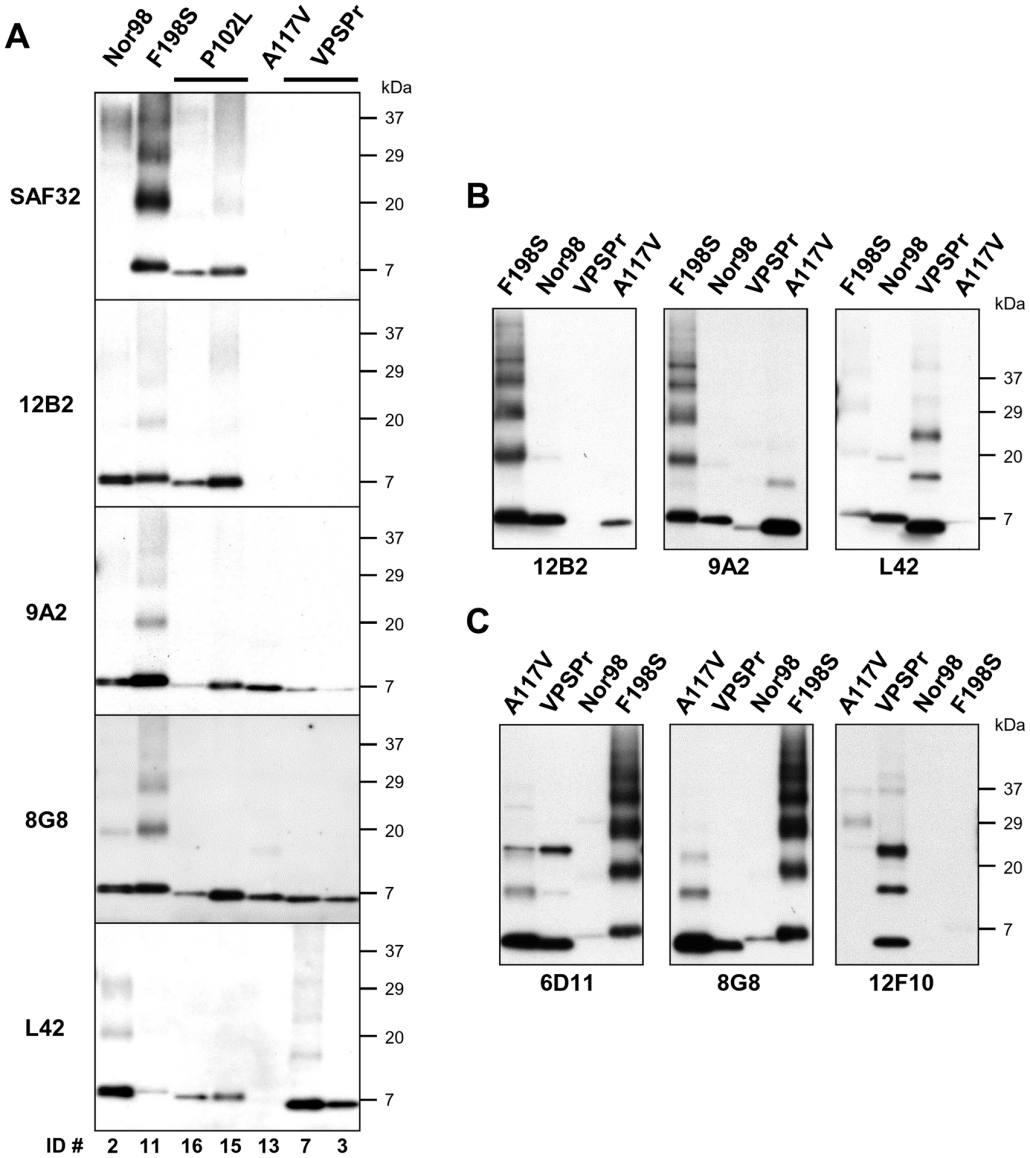
Epitope mapping analysis. **A:** Representative blots of PrP^res^ from Nor98, GSS and VPSPr. Brain homogenates were treated with 100 µg/ml PK. Replica blots were probed with SAF32, 12B2, 9A2, 8G8, L42 mAbs. Case numbers are shown on the bottom of blots according to Table 1. **B:** Samples (ID #11, #1, #7, #13) were re-analysed at a lower PK concentration (50 µg/ml) to investigate partially cleaved epitopes. Replica blots were probed with 12B2, 9A2 and L42 mAbs. In these blots Nor98 is an internal control as it is recognised equally well by the 3 mAbs (see Fig. 4A). By comparison with Nor98, it is shown than i) in GSS A117V the L42 epitope is cleaved, the 12B2 is partially preserved and the 9A2 was fully preserved; ii) in VPSPr the 12B2 epitope is cleaved and the 9A2 partially preserved; iii) in GSS F198S the L42 epitope is partially preserved. **C:** Samples (ID #11, #1, #7, #13) were treated with 100 µg/ml PK and probed with 6D11, 8G8 and 12F10 mAbs. 6D11 and 8G8 bind to epitopes within the “core” of PrP^res^ fragments and reveal the actual quantity of PrP^res^ fragments in the samples analysed. By comparison, 12F10, which recognizes an epitope at the C-terminus of PrP^res^ fragments, only detected VPSPr, suggesting that its epitope is cleaved in all other samples.

Direct comparison of the gel mobility of PrP^res^ fragments showed that the apparent MW in VPSPr and GSS A117V was consistently lower than that of the matching fragments in Nor98, GSS F198S and GSS P102L (Fig. 4). The apparent MW was between 7 and 8 kDa in GSS F198S, slightly lower in Nor98 and GSS P102L, and less than 7 kDa in VPSPr and GSS A117V.

Only 3 mAbs (8G8, 6D11 and F89), with epitopes between aa 97 and 142, detected equally well all PrP^res^ species (Fig. 4, Table 2). The epitopes recognized by 8G8 and 6D11 extend towards the C-terminus compared with that of the overlapping 9A2, and include at least in part the central polybasic domain (PBD) of PrP [64]. Accordingly, the 9A2 epitope was also detected in all PrP^res^ species, although in VPSPr the binding of 9A2 was greatly lower compared with other mAbs (Fig. 4A and 4B). PK titration of VPSPr analyzed by 9A2 and L42 confirmed a preferential trimming of 9A2 epitope with increasing PK concentrations (Fig. 3). These findings imply that the N-terminal PK cleavage in VPSPr involved at least in part the 99-WNK-101 epitope, but did not extend to the nearby PBD. The 100–142 PrP peptide, which includes the PDB and the highly conserved hydrophobic domain, as well as the endogenous proteolytic α-cleavage site, were thus common to all PrP^res^ internal fragments.

Antibodies SAF32, 12B2 and 9A2 contributed to define all others N-terminal PK cleavage sites. GSS A117V was negative with SAF32 and 12B2, but positive with 9A2 (Fig. 4A), implying N-terminal cleavage between aa 93 and 99. However, we previously observed that at intermediate PK concentrations the 12B2 epitope was partially spared (Fig. 3). Further analyses at 50 µg/ml PK clearly showed that in GSS A117V the 12B2 epitope was partially preserved and the 9A2 was fully preserved (Fig. 4B). The N-terminus of GSS F198S and P102L included at least part of the octarepeat region, being detected by SAF32, which recognizes a repeated epitope within the octarepeat region (OR), and by 12B2 (Fig. 4A). Similarly, in Nor98 the N-terminus extended towards the OR, being strongly detected by 12B2 (Fig. 4) and P4 (Fig. 1), whose epitopes lie at the C-terminus of the OR and partially overlap with the last epitope of SAF32. Notwithstanding, SAF32 did not bind to Nor98 PrP^res^, although the meaning of this finding is unclear, due to a G92 insertion in sheep PrP which could have interfered with the binding of SAF32 to the last repeat (Fig. S3).

At the C-terminus, none of the PrP^res^ fragments comprised the epitope of SAF60 (Table 2), implying a PK cleavage before aa ∼160 in all PrP^res^ species. The 12F10 epitope was detected in VPSPr cases only (Fig. 4C), while the L42 epitope was present in Nor98, VPSPr and GSS P102L, barely detectable in GSS F198S and cleaved in GSS A117V (Figs. 4A and 4B). Given that 12F10 extends 2 residues towards the C-terminus compared to L42 (aa 150 *vs* 152), these findings imply that the C-terminal PK cleavage is between aa 152 and 157 in VPSPr, between aa 150 and 152 in Nor98 and GSS P102L, and between aa 142 and 150 in GSS F198S and GSS A117V. Direct comparison of GSS F198S and GSS A117V at 50 µg/ml PK confirmed that the L42 epitope was preferentially cleaved in GSS A117V compared to GSS F198S (Fig. 4B), in line with previous observations in PK titration experiments (Fig. 3).

**Figure 5:**
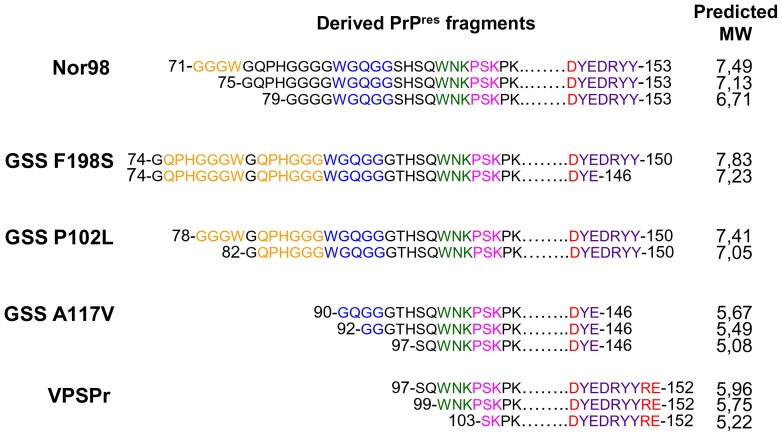
Comparison of PrP^res^ fragments in Nor98, GSS and VPSPr. The aligned amino acid sequences at the N and C termini of all PrP^res^ fragments, derived from epitope mapping as described in SI 5, are depicted. Amino acid numbering refers to sheep PrP for Nor98 and to human PrP for all other fragments. Coloured letters highlight the relevant epitopes as follows: orange for SAF32; blue for 12B2; green for 9A2; pink for 8G8; red for 12F10 and violet for L42. Note that when epitopes of mAbs partially overlap, only the aa differentiating the epitopes were coloured. On the right of each PrP^res^ fragment is reported the predicted MW estimated by using the ProtParam (ExPASy) software.

Based on the data described above and summarised in Table 2, and on the available data on PK cleavage sites in human and sheep PrP^Sc^ (Fig. S3), we derived the PrP peptides forming the protease-resistant core of the different PrP^Sc^ studied (Fig. 5). Importantly, the predicted molecular masses (Fig. 5) closely matched the rank of apparent MW observed by WB. Indeed, VPSPr and GSS A117V had the smallest PrP^res^ fragments, with predicted MW of 5.2–5.9 kDa and 5.1–5.7 kDa. Despite sharing the same MW, VPSPr and GSS A117V showed different C- and N-terminal PK cleavages, so that the VPSPr PrP^res^ was C-terminally shifted compared to GSS A117V. All other PrP^res^ fragments were 1.5–2 kDa higher, with predicted MW of 6.7–7.5 kDa in Nor98, 7–7.4 kDa in GSS P102L, and 7.2–7.8 kDa in GSS F198S. The N-terminal PK cleavage site gave a major contribution to the observed MW variability, with the variably cleaved N-terminal PrP region comprising the type 1 and type 2 N-terminal cleavages described in sCJD [67].

Overall, detailed epitope mapping revealed that i) VPSPr and GSS had different protease resistant cores of PrP^Sc^, ii) in GSS, the protease resistant core varied according to the PrP mutation, and iii) the protease resistant core of Nor98 largely overlapped with that of GSS P102L.

### Conformational Stability of PrP^Sc^ from Nor98, VPSPr and GSS

**Figure 6:**
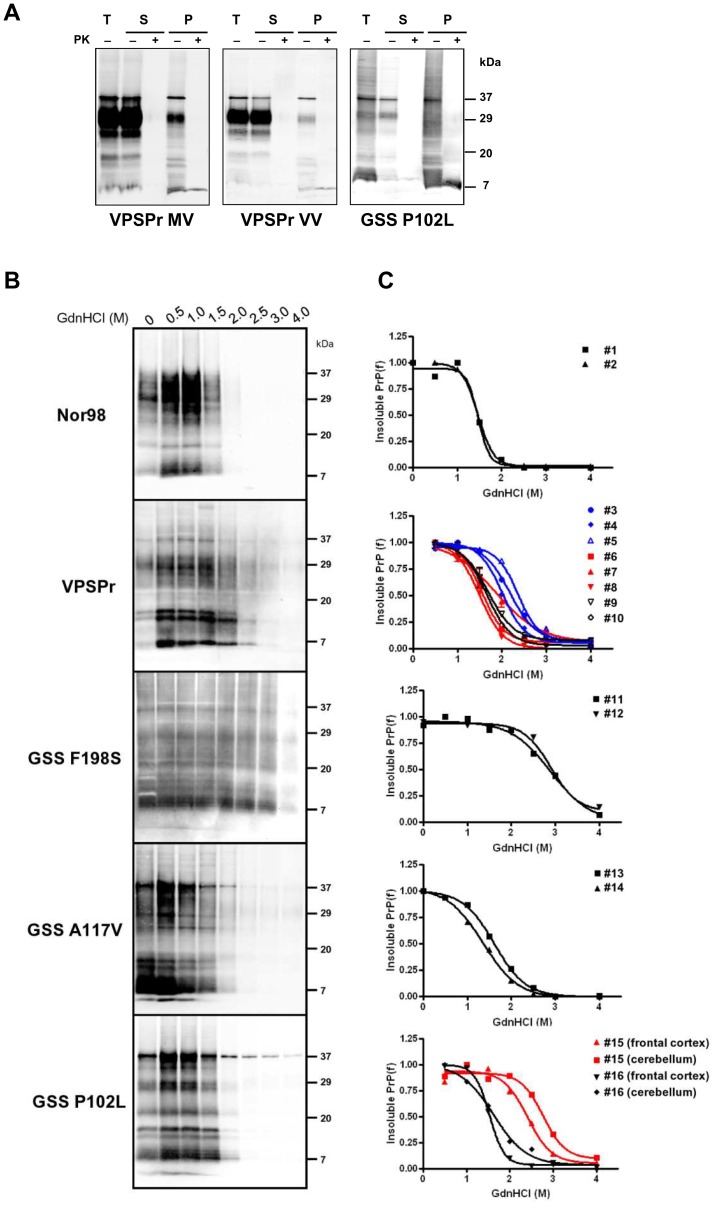
Conformational stability of insoluble PrP^Sc^. **A:** Western blot analysis of VPSPr 129MV (#7) and 129VV (#3) and GSS P102L (#15 cerebellum) cases, showing separation of insoluble PrP^Sc^ by a solubility assay. Samples were centrifuged at 20000 g for 1 h in presence of 1% sarkosyl. Supernatant (S) and pellet (P) fractions were analysed with (+) or without (-) PK treatment (50 µg/ml), along with aliquots of samples before centrifugation (Tot). Note that PrP^res^ segregated into the insoluble fraction (compare lanes S+ and P+ in each blot). In each lane 0,7 mg TE were loaded. Membranes were probed with L42 mAb. **B:** Representative western blots of CSSA experiments in Nor98 (#1), VPSPr (#9), GSS F198S (#11), GSS A117V (#14) and GSS P102L (#16) cases. Lanes were loaded with insoluble PrP obtained as shown in Fig. 6A, with or without previous denaturation with increasing concentrations of GdnHCl, as shown on the top of each lane. Membranes were probed with L42. Molecular size markers are shown in kilodaltons on the right of each blot. **C:** Dose-response curves obtained by plotting the fraction of PrP^Sc^ remaining in the pellet as a function of GdnHCl concentration and best-fitted and using a four parameter logistic equation. Individual [GdnHCl]_1/2_ values are shown in Table 1.

We have recently shown that insoluble PrP^Sc^ in Nor98 is mostly sensitive to PK compared to insoluble PrP^Sc^ in classical forms, such as scrapie and sCJD [58]. By using the same solubility assay, we found that VPSPr and GSS were similar to Nor98 in this respect (Fig. 6A). Indeed, although protease resistant PrP^Sc^ segregated in the insoluble fraction, most of the insoluble PrP^Sc^ was sensitive to PK (Fig. 6A). The conformational properties of this insoluble fraction containing both, protease sensitive and protease-resistant PrP^Sc^ species were then studied by a new assay, that we named conformational stability and solubility assay (CSSA) [58]. CSSA derives the conformational stability of PrP^Sc^ aggregates by measuring their solubility under increasing concentrations of guanidine hydrochloride. We thus determined GdnHCl_1/2_ values for all individual samples by CSSA (Table 1, Fig. 6B). In line with previous observations [58], Nor98 cases were sensitive to guanidine denaturation, with GdnHCl_1/2_ values of 1.4 and 1.5 M. Unexpectedly, the conformational stability was quite variable among VPSPr cases, with GdnHCl_1/2_ values between 1.5 M and 2.4 M. This variability may be associated with at least in part by the codon 129 genotype, as 129MM cases and 129MV cases were less stable (range 1.5–1.9 M) than 129VV cases (range 2.0–2.4 M). In contrast, despite having different PrP genotypes at codon 129, GSS F198S cases showed overlapping denaturation profiles, being remarkably stable (2.9 M). A similar observation was made with A117V cases, which however showed a dramatically lower conformational stability compared to F198S (1.5 vs 2.9 M). Finally, the 2 GSS cases with P102L mutation gave divergent results: the case with 8 kDa PrP^res^ alone was sensitive to guanidine denaturation (1.5–1.6 M), while the case with PrP^27–30^ and 8 kDa PrP^res^ was much more stable (2.4–2.8 M), independently on the brain area analysed (Table 1).

## Discussion

With the appearance of the BSE agent, the boundaries separating animal from human prion diseases have been crossed for the first time. From then on, the risk of animal prion diseases for human beings has become a crucial matter of investigation. Here we have analyzed a group of atypical prion diseases that have never been the subject of comparative studies at the animal-human interface. We used refined biochemical techniques that allowed to derive the conformational properties of PrP^Sc^ aggregates in well known, and newly discovered human prion diseases, such as GSS and VPSPr, in comparison with small ruminants Nor98. Our results indicate that PrP^Sc^ from these atypical TSEs share remarkable biochemical features that distinguish them from PrP^Sc^ of classical TSEs, such as sCJD and classical scrapie. Indeed, we found that GSS, VPSPr and Nor98 were characterized by i) high levels of insoluble, but mainly PK sensitive PrP^Sc^, ii) variable quantities of a strongly PK resistant PrP^Sc^ component, and iii) a protease resistant core made up of internal PrP^res^ fragments with ragged N and C termini. Furthermore, in several instances it was possible to observe the presence of mild and strong PrP^res^ fragments also in PK titration studies. Until recently, such biochemical features were exclusively associated with PrP^Sc^ aggregates characteristic of some phenotypic subtypes of GSS [21]–[23], [26], [68], which contained mainly mutant PrP alleles [21], [25], [28]–[30]. In this study, the same atypical features were found to characterise PrP^Sc^ from other sporadic human and animal TSEs co-analysed with GSS subtypes, well distinguishable from classical types of PrP^Sc^ found in CJD and scrapie. Indeed, when analysed with similar approaches in previous studies, CJD and classical scrapie didn’t show evidence of being enriched in insoluble and protease sensitive PrP^Sc^ [58], [69]. Furthermore, although PK cleavages other than those at residues 82 and 97 have been reported in sCJD [67], scrapie [70] and H-type BSE [71], in all these instances the glycosylated C-terminus of PrP^Sc^ was always part of the protease resistant core. Despite being epitomized by common biochemical features, atypical PrP^Sc^ types also showed an unexpected molecular complexity, which was reminiscent of the heterogeneity observed among classical PrP^Sc^ types. Indeed, the atypical PrP^Sc^ types studied encompassed the same range of conformational stabilities previously observed in classical PrP^Sc^ types [58], [60], [69], [72], [73], and the N-termini of PrP^res^ spanned the same differential PK cleavages which discriminate sCJD types 1 and 2 [67], as well as scrapie and BSE [74], [75].

In line with the finding that a single prion strain has been isolated from Nor98 cases [47], [53], [76] and with previous observations by CSSA [58], we found homogenous biochemical properties by PrP^res^ typing of several Nor98 isolates, irrespectively from the species and the PrP genotype. In the present and previous studies [54], [55] using harsh PK conditions, the biochemical signature of Nor98 was a strongly PK resistant PrP^res^ with ragged N and C termini, at variance with the findings obtained with mild PK protocols [51]. Our findings in PK titration experiments showed indeed that mild and strong PrP^res^ co-exist in Nor98. Despite mild PK digestion might assure diagnostic sensitivity for this particular PrP^Sc^ type, our results suggest that such protocols could not be optimal for strain typing, and might lead to detect PrP fragments derived from incomplete proteolysis or to overestimate individual differences in WB patterns. Indeed, PK titration experiments showed that partially PK resistant fragments were sequentially produced and trimmed within a very small range of PK concentrations, so that the labile nature of these PrP fragments makes them susceptible to slight variations of the experimental conditions or even of their preservation.

Whereas biochemical techniques allowing direct investigation of PrP^Sc^ conformation are lacking, the issue of deriving conformational properties of PrP^Sc^ aggregates by indirect approaches, such as the study of PrP^res^ fragments by immunoblotting, was made even more challenging by i) the need to compare PrP^Sc^ from different species which have different PrP primary structures, and ii) the presence of conspicuous amounts of partially protease sensitive PrP^Sc^ in the prion diseases under investigation, whose labile nature undermines the use of protease-based techniques. We believe that our overall approach was able to successfully tackle these drawbacks, allowing for the first time a thorough biochemical characterization of atypical PrP^Sc^ types in human and animals. Indeed, for the epitope mapping of strong PK-resistant PrP^res^ fragments we selected a panel of antibodies able to recognise non-conformational epitopes conserved in human and small ruminants, which allowed to define the N- and C-terminal PK cleavage sites of PrP^Sc^. In order to ensure the elimination of mild PrP^res^ fragments, whose incomplete proteolysis might have interfered with the epitope-mapping results, the PK concentration was chosen on the basis of PK titration curves. Furthermore, by showing sequential trimming of N- and C-terminal epitopes, PK titration experiments provided an independent proof that the absence of binding of a given antibody at high PK concentration was actually due to the epitope trimming by PK and not to unrelated confounding factors. Importantly, direct sequencing of PK-treated PrP^res^ fragments in patients with P102L, A117V and F198S mutations have been reported [21], [23], thus allowing to validate our epitope mapping approach. Indeed, N-terminal sequence and mass spectrometry demonstrated that the 7 kDa PrP^res^ fragment of P102L was constituted by aa 78–82 to 147–153 [21], in very good agreement with our determination of aa 78–82 to 150 (see Fig. 5). In A117V and F198S, the N-termini were aa 90 and 74 [23], very similar to those we found by epitope mapping, i.e. aa 90–96 and 74, respectively (see Fig. 5).

On the other side, for deriving conformational information on protease sensitive PrP^Sc^, we took advantage of a new technique, the CSSA, that we have previously shown being able to measure the conformational stability of both protease sensitive and protease resistant PrP^Sc^, without any protease step [58]. Importantly, CSSA was equally applicable to different species and was shown to discriminate human sCJD strains and small ruminant TSEs, either in the original species and after transmission in bank voles [58], [60]. As such, CSSA represents the only available biochemical approach for comparing the conformational stability of PrP^Sc^ from different species.

It has been previously suggested that PrP^Sc^ from GSS patients with different PrP mutations may have distinct conformations, owing to their different N-terminal cleavages [23]. Our results confirm and extend these findings, in that they showed heterogeneity among GSS cases in the C-terminal cleavage of PrP^res^, too. Overall, A117V, F198S and P102L mutations were associated with three distinct PrP^res^ fragments. The finding that A117V and F198S were also discriminated by highly different PrP^Sc^ conformational stabilities further corroborates the hypothesis that the distinct PK cleavage sites actually reflect different PrP^Sc^ conformations, possibly representing different prion strains.

Interestingly, while the two P102L cases investigated had identical PrP^res^ fragments, they were discrepant by CSSA, with the case exhibiting the 7–8 kDa PrP^res^ alone being much more susceptible to denaturation compared to the one with PrP^27–30^. The higher stability of the latter case was confirmed in brain areas in which PrP^27–30^ could not be detected (see Fig. S2), suggesting a major role of protease sensitive PrP^Sc^ in determining the observed difference in conformational stability. Although further studies on a larger panel of samples are needed to corroborate this finding, it is worth noting that closely similar results have been recently reported by using an alternative approach, based on 3F4 epitope exposure in insoluble PrP^Sc^ by concentration-dependent guanidine denaturation, with GdnHCl_1/2_ values of 2.4 M and 1.9 M for P102L cases characterized by having PrP^27–30^ or not [69]. As the presence of either PrP^27–30^ or 7–8 kDa correlates with distinctive pathologic [21], [22] and infectious [77] features in P102L cases, the finding that they might be endowed with different conformational variants of PrP^Sc^ would not be surprising.

When VPSPr was first described, the similarities with GSS led to hypothesize that VPSPr could represent the sporadic counterpart of GSS [31], [32]. In our study, VPSPr cases did not overlap with any of the PrP^Sc^ phenotypes observed in GSS, thus suggesting that VPSPr is a distinct pathological entity. Indeed, PrP^res^ in VPSPr was C-terminally shifted compared to all GSS types, including A117V which shared with VPSPr the same apparent MW. Accordingly, the signatures able to discriminate VPSPr from GSS PrP^Sc^ were the presence of the 12F10 epitope but the absence of the 12B2 epitopes. Whereas the ∼6 kDa fragment was identical in all VPSPr cases, we were surprised to find a quite strong conformational heterogeneity by CSSA, in large part explained by the genotype at PrP codon 129, as 129VV cases were more resistant to GdnHCl denaturation compared to 129MV and 129MM. Interestingly, previous studies showed clinical and biochemical differences among the 3 genotypes, with 20 and 26 kDa mild PrP^res^ fragments being more susceptible to PK in 129VV than in 129MV and 129MM [32]. Taken together, these findings suggest that the observed heterogeneity among VPSPr cases could be mainly due to protease sensitive PrP^Sc^ components. Whether these differences simply reflect different molecular constraints posed by M or V at residue 129, or imply more fundamental differences in transmissibility and possibly strain features, is currently under investigation.

Overall, our data suggest that atypical prions encompass PrP^Sc^ types with remarkable conformational diversity, mirrored by different protease resistant cores and conformational stabilities. The combined results of epitope mapping and CSSA allowed us to discriminate human samples according to their diagnosis and PrP mutation, suggesting that atypical PrP^Sc^ conformations are indeed disease-specific. Taking into consideration the observed heterogeneity and the discriminatory power of our approach, it seems remarkable that the PrP^Sc^ phenotype of Nor98 largely overlapped with GSS P102L characterised exclusively by 7–8 kDa PrP^res^. Such a biochemical “closeness” raises the question of a possible strain identity between GSS P102L and Nor98. Given that the biochemical approach we used does not characterize the biological properties of the prions studied, the strain properties of GSS and Nor98 are currently under investigation by bioassay in rodent models.

At present the only epidemiological link between animal and human TSEs has been demonstrated for classical BSE and variant CJD [16], [78], showing for the first time the zoonotic potential of TSEs. Since then, the implementation of active surveillance in livestock has led to the identification of Nor98 and other previously unrecognised animal prion strains, mainly with a sporadic occurrence, whose origin and zoonotic potential are still poorly understood [79]. It has been previously shown that peripheral tissues of sheep with Nor98 might harbour detectable levels of infectivity [49], [50], indicating that infectious material might enter the food chain. On the other hand, the well known genetic aetiology of GSS suggests that the similar PrP^Sc^ conformations found in Nor98 and GSS P102L are unlikely to indicate a common infectious source, but might derive from a similar molecular mechanisms involved in PrP^C^-to-PrP^Sc^ conversion.

Finally, in GSS P102L cases with PrP^27–30^ and 7–8 kDa PrP^res^, PrP^27–30^ was reported to correlate with the presence of spongiform degeneration and synaptic PrP deposition, whereas the 7–8 kDa fragment was associated with multicentric amyloid deposits in brain regions with little or no vacuolation [21], [24], arguing for a low toxicity of these PrP^Sc^ aggregates. Furthermore, the few successful attempts to transmit GSS P102L derived from the inoculation of cases with PrP^27–30^ [77], [80], [81]. In contrast, inoculation of cases characterized exclusively by 7–8 kDa PrP^res^ resulted in the absence of disease transmission even in transgenic mice carrying the P101L mutation, but elicited PrP-amyloid deposition in several recipient mouse brains [77]. Our finding that Nor98 shares PrP^Sc^ conformation with 7–8 kDa GSS P102L, together with the reported easy of transmissibility of Nor98 in several animal models [47], [49], [50], [76], [82], [83], challenges the view that PrP^Sc^ with a 7–8 kDa protease-resistant core is *per se* associated with little or no transmissibility, and deserves further investigation by comparative transmission studies of Nor98 and GSS in animal models known to be susceptible to Nor98.

## Materials and Methods

### Brain Tissues

Small ruminant brain tissues were from Italian and Norwegian field cases, detected by active and passive surveillance, and are described in Table S1. Patients with VPSPr, and GSS were referred to the National Prion Disease Pathology Surveillance Center, Cleveland, OH. Their individual features, including diagnosis, PrP polymorphisms and mutations, and the brain areas sampled for analysis, are reported in Table 1. Coronal sections of human brain tissues were obtained at autopsy and stored at −80°C until use. Written consent to use human autopsy material for research purposes had been obtained from patients or legal guardians for all samples. Clinical data and relevant hospital records were coded and handled according to the protocols approved by the Ethical Committee and Institutional Review Board of Case Western Reserve University to protect patients’ identities. This retrospective study was approved by Ethical Committee and Institutional Review Board of Case Western Reserve University. Brain material from sheep and goat with Nor98 was obtained in Italy and Norway from official surveillance activities conducted according to EU Regulation 999/2001 and the ethics committee approval was not necessary.

### Anti-PrP Antibodies

The antibodies used for comparative epitope mapping in human and sheep and their epitopes are reported in Table 2 or diagrammatically represented in Fig. 5. Other antibodies used were: P4 (sheep PrP residues 93–99), 6C2 (human PrP residues 106–119), SAF84 (sheep PrP residues 167–173), 1E4 (human PrP residues 97–105) [84], 3F4 (human PrP residues 106–112) [85]. 9A2 and 12B2 mAbs were kindly provided by Dr. Jan Langeveld (Central Veterinary Institute of Wageningen, Lelystad, Netherlands).

### PrP^res^ Analysis

Typing of PrP^res^ was performed by discriminatory immunoblotting, according to the ISS discriminatory WB method (http://www.defra.gov.uk/vla/science/docs/sci_tse_rl_handbookv4jan10.pdf). Briefly, brain homogenates (20% w/v) were prepared in 100 mM Tris-HCl with Complete protease inhibitor cocktail (Roche, Indianapolis, IN, USA) at pH 7.4. The homogenates were either used directly or stored at −20°C. After adding an equal volume of 100 mM Tris-HCl containing 4% sarkosyl, the homogenates were incubated for 30 min at 37°C with gentle shaking; proteinase K (0 to 1 mg/ml; Sigma-Aldrich, St. Louis, MO, USA) was added, and then the homogenates were further incubated for 1 h at 37°C with gentle shaking. For epitope mapping a final concentration of 100 µg/ml was used. The reaction was stopped with 3 mM PMSF (Sigma-Aldrich, St. Louis, MO, USA). The samples were added with an equal volume of isopropanol/butanol (1:1 v/v) and centrifuged at 20000 g for 5 min, the supernatants were discarded and the pellets were dissolved in denaturing sample buffer for WB analysis.

### Conformational Stability and Solubility Assay

As previously described [58], the conformational stability was analysed by CSSA. Briefly, aliquots of brain homogenates (6% w/v in 100mM TrisHCl, pH 7.4) were added with an equal volume of 100 mM TrisHCl (pH 7.4), sarcosyl 4% and incubated for 1h at 37°C with gentle shaking. Then, aliquots of each sample were treated (1 h at 37°C) with different concentrations of GdnHCl (Pierce) to obtain final concentrations ranging from 0 to 4 M. After GdnHCl treatment samples were centrifuged at 20000 g for 1 h at 22°C. Pellets were re-suspended in 1× Laemmli sample buffer (Bio-Rad Laboratories, Hercules, CA) and 10% β-mercaptoethanol (Bio-Rad Laboratories, Hercules, CA) and analysed by WB. The dose-response curves allowed to estimate the concentration of GdnHCl able to solubilise 50% of PrP^Sc^ (GdnHCl_1/2_). Individual denaturation curves were analyzed and best-fitted by plotting the fraction of PrP^Sc^ remaining in the pellet as a function of GdnHCl concentration, and using a four parameter logistic equation (GraphPad Prism).

### Western Blot

For the initial analyses of sheep samples (Fig. 1 and Fig. S1), WB was performed following the procedures described in ISS discriminatory WB [56], [59], [60], by using 12% bis-Tris polyacrylamide gels (Invitrogen) for electrophoresis, and the VersaDoc imaging system (Bio-Rad) for PrP visualization. For all other WB experiments, pellets were re-suspended in 1× Laemmli buffer (Bio-Rad Laboratories, Hercules, CA) and 10% β-mercaptoethanol (Bio-Rad Laboratories, Hercules, CA) and loaded onto 15% Tris-HCl Criterion precast gels (Bio-Rad Laboratories, Hercules, CA) for gel electrophoresis and Western blotting. Proteins were transferred to Immobilon-P polyvinylidene fluoride membrane (Millipore, MA, USA) for 2 hours at 70V. The blots were blocked in PBS containing 0.05% Tween 20 and 1% non-fat milk powder for 1 h, and then incubated for 1 hour at room temperature with anti-PrP antibodies. Following incubation with horseradish peroxidase-conjugated antimouse immunoglobulin G (IgG) at 1:12,000 for 1h, the PrP bands were visualized by ECL Plus (Amersham Biosciences, Piscataway, NJ, USA) on Kodak film (Eastman Kodak, Rochester, NY). For quantitative purposes, CSSA samples were transferred to Immobilon-FL membrane (Millipore, MA, USA) and blocked with Odyssey Blocking Buffer (LI-COR Biosciences, Lincoln, NE, USA). The primary antibody was diluted in Odyssey Blocking Buffer plus 0.1% Tween-20) and IRDye800CW (LI-COR Biosciences) was used as fluorescently-labelled secondary antibody (diluted in Odyssey Blocking Buffer plus 0.1% Tween-20 and 0.01 SDS). The incubations with antibodies were performed for 1 h at room temperature with gentle shaking, and after each incubation the membranes were washed 4×5 min in PBS with 0.1% Tween-20. Next the membranes were scanned for infrared signal using Odyssey Imaging System (LI-COR Biosciences) and the signal intensities were analyzed by using the Odyssey infrared image system software (Li-Cor).

## Supporting Information

Figure S1 **Characterization of Nor98 PrP^res^ fragments.** Comparative PK titration in Nor98 and classical scrapie. PK digestion curves of classical scrapie (n = 4) and Nor98 (n = 4) with concentrations of PK ranging from 0.006 to 6.4 mg/ml. Replica blots were probed with SAF84 and P4 and selected fragments were quantified. Representative WB are shown in the left panels, and the quantitative analysis of PrP^res^ for the determination of the PK_1/2_ on the right panels. For quantitative purposes, both high and low MW PrP^res^ were measured in Nor98, while the unglycosylated PrP^27–30^ was measured in scrapie. In Nor98 (upper panels), there was a clear-cut distinction between fragments with different resistance to proteolysis. High MW fragments displayed very low PK_1/2_, ranging from 0.01 to 0.03 mg/ml, while the P4-positive low MW fragments were still detected at the highest PK-concentrations. Low MW PrP^res^ amount increased up to 0.025 mg/ml PK and then declined, similarly to what observed with PrP^27–30^ in classical scrapie. The PK_1/2_ of low MW PrP^res^ ranged from 0.21 to 0.53 mg/ml PK. In classical scrapie (lower panels), the levels of PrP^27–30^ reached a plateau between 0.012 and 0.05 mg/ml PK with P4 and between 0.012 and 0.2 mg/ml PK with SAF84; afterwards the quantity of PrP^27–30^ declined with both mAbs. Interestingly, the decline of SAF84-positive PrP^res^ was parallel but shifted to the right compared to that measured by P4. Quantitative analysis showed that the PK_1/2_ for degradation of PrP^27–30^ ranged from 0.24 to 0.68 mg/ml with P4 and from 1 to 2.1 mg/ml with SAF84.
(TIF)

Figure S2 **PrP^res^ phenotypes in GSS P102L cases.** Western blot of the two GSS P102L cases (#15 and #16, Table 1). Samples were treated with 50 µg/ml PK and membranes were probed with F89. MW markers are shown in kilodaltons on the left.
(TIF)

Figure S3 **Derivation of the N and C terminal PK cleavage sites from epitope mapping data.** The PK cleavages were derived taking into account the epitope mapping data, summarised in Table 2, the known N-terminal cleavage sites in sCJD [67] and in scrapie or sheep BSE [74], [75], and the potential cleavage sites cleaved by PK in the corresponding human and sheep PrP sequences, as predicted by the PeptideCutter software (ExPASy). The cleavage sites used for our determination are represented by arrows on the top of the human and sheep aa sequences, while the derived cleavage sites for GSS, VPSPr and Nor98 are represented by arrows below the aa sequences. Coloured letters highlight the epitopes of the relevant mAbs (reported in the figure with the corresponding colour) used in epitope mapping experiments. When epitopes of mAbs partially overlap, only the aa differentiating the epitopes were coloured (for a full description of epitopes see Table 2). For the sake of clarity, in all instances where two consecutive amino acids where deemed as possible cleavage sites, only one of them was reported in the figure. At the N-terminus (upper panel), VPSPr PrP^res^ did not included SAF32 and 12B2 epitopes and only partially included the 9A2 epitope, and thus the derived cleavage sites were those before and after the 9A2 epitope, i.e. S97, W99 and S103, corresponding to cleavage sites detected in type 2 sCJD. GSS A117V PrP^res^ included the 9A2 epitope, but not completely the 12B2, with derived cleavage sites G90, G92 and S97, corresponding to cleavage sites detected in type MV2 and VV2 sCJD. For the other PrP^res^ types having 12B2 and SAF32 epitopes it was less obvious to derive potential cleavage sites. Indeed, SAF32 recognises an epitope repeated 4 times within the OR sequence, so that for SAF32-positive samples could have been cleaved at several positions within the OR sequence. However, based on the N-terminal sequencing reported for sCJD and scrapie and on the apparent MW observed in WB, we considered highly likely that GSS P102L PrP^res^ contained only one SAF32 epitope, with cleavages derived at G78 and G82. GSS F198S repeatedly showed an apparent MW slightly higher than GSS P102L and was detected by SAF32 with higher sensitivity compared with GSS P102L (see Fig. 4), the most likely explanation being that the N-terminus of GSS F198S contained two SAF32 epitopes, corresponding to cleavage at G74. In sheep, due to a G92 insert mutation within the last repeated epitope recognised by SAF32, it is probable, although not proven, that SAF32 only bind with high sensitivity to three repeated epitopes. To make evident this difference between human and sheep sequences, the putative last SAF32 epitope in sheep was not highlighted in red. Although we were not able to find any alternative antibody which recognised a repeated epitope within the OR not encompassing the sheep insert mutation, which would have allowed a more precise definition of the N-terminus of Nor98 PrP^res^, based on the apparent MW, which was similar or slightly higher than that of P102L PrP^res^, and on the presence of the aa 93–97 epitope also after strong PK treatment, the simplest interpretation of the data is that the N-terminus of Nor98 PrP^res^ extends to the last octarepeat, as it was for GSS P102L. However, a more C-terminal cleavage at G86 was also derived because, for the arguments described above, it was not formally possible to exclude it. At the C-terminus, all PrP^res^ types lacked the SAF60 epitope and only VPSPr PrP^res^ was positive with 12F10. Consequently, the cleavage site for VPSPr was derived at E152, the last predicted PK cleavage site before Y157 which would have disrupted the SAF60 epitope. The only possible cleavage derived for GSS P102L and Nor98 was E150, being both positive with L42 and negative with 12F10. GSS F198S partially lost the L42 epitope, with derived PK cleavages at Y145 or E146 within the L42 epitope, and E150 which leaves intact the L42 epitope. Finally, GSS A117V PrP^res^ was not bound by L42 but included the F89 epitope, with resulting potential cleavage at Y145 or E146.
(TIF)

Table S1 Table with sheep and goat samples.
(DOC)

## References

[1] Bishop MT, Will RG, Manson JC (2010) Defining sporadic Creutzfeldt-Jakob disease strains and their transmission properties. Proc Natl Acad Sci U S A 107: 12005–12010. 20547859 10.1073/pnas.1004688107 PMC2900653

[2] Korth C, Kaneko K, Groth D, Heye N, Telling G, et al (2003) Abbreviated incubation times for human prions in mice expressing a chimeric mouse-human prion protein transgene. Proc Natl Acad Sci U S A 100: 4784–4789. 12684540 10.1073/pnas.2627989100 PMC153633

[3] Mastrianni JA, Nixon R, Layzer R, Telling GC, Han D, et al (1999) Prion protein conformation in a patient with sporadic fatal insomnia. N Engl J Med 340: 1630–1638. 10341275 10.1056/NEJM199905273402104

[4] Nonno R, Di Bari MA, Cardone F, Vaccari G, Fazzi P, et al (2006) Efficient transmission and characterization of Creutzfeldt-Jakob disease strains in bank voles. PLoS Pathog 2: e12. 16518470 10.1371/journal.ppat.0020012 PMC1383487

[5] Parchi P, Cescatti M, Notari S, Schulz-Schaeffer WJ, Capellari S, et al (2010) Agent strain variation in human prion disease: insights from a molecular and pathological review of the National Institutes of Health series of experimentally transmitted disease. Brain 133: 3030–3042. 20823086 10.1093/brain/awq234 PMC2947429

[6] Bruce ME, Boyle A, Cousens S, McConnell I, Foster J, et al (2002) Strain characterization of natural sheep scrapie and comparison with BSE. J Gen Virol 83: 695–704. 11842264 10.1099/0022-1317-83-3-695

[7] Pattison IH, Millson GC (1961) Scrapie produced experimentally in goats with special reference to the clinical syndrome. J Comp Pathol 71: 101–109. 13733383 10.1016/s0368-1742(61)80013-1

[8] Beringue V, Andreoletti O, Le Dur A, Essalmani R, Vilotte JL, et al (2007) A bovine prion acquires an epidemic bovine spongiform encephalopathy strain-like phenotype on interspecies transmission. J Neurosci 27: 6965–6971. 17596445 10.1523/JNEUROSCI.0693-07.2007 PMC6672218

[9] Beringue V, Bencsik A, Le Dur A, Reine F, Lai TL, et al (2006) Isolation from cattle of a prion strain distinct from that causing bovine spongiform encephalopathy. PLoS Pathog 2: e112. 17054396 10.1371/journal.ppat.0020112 PMC1617128

[10] Biacabe AG, Laplanche JL, Ryder S, Baron T (2004) Distinct molecular phenotypes in bovine prion diseases. EMBO Rep 5: 110–115. 14710195 10.1038/sj.embor.7400054 PMC1298965

[11] Buschmann A, Gretzschel A, Biacabe AG, Schiebel K, Corona C, et al (2006) Atypical BSE in Germany–proof of transmissibility and biochemical characterization. Vet Microbiol 117: 103–116. 16916588 10.1016/j.vetmic.2006.06.016

[12] Casalone C, Zanusso G, Acutis P, Ferrari S, Capucci L, et al (2004) Identification of a second bovine amyloidotic spongiform encephalopathy: molecular similarities with sporadic Creutzfeldt-Jakob disease. Proc Natl Acad Sci U S A 101: 3065–3070. 14970340 10.1073/pnas.0305777101 PMC365745

[13] Prusiner SB (1991) Molecular biology of prion diseases. Science 252: 1515–1522. 1675487 10.1126/science.1675487

[14] Bessen RA, Marsh RF (1992) Biochemical and physical properties of the prion protein from two strains of the transmissible mink encephalopathy agent. J Virol 66: 2096–2101. 1347795 10.1128/jvi.66.4.2096-2101.1992 PMC289000

[15] Caughey B, Raymond GJ, Bessen RA (1998) Strain-dependent differences in beta-sheet conformations of abnormal prion protein. J Biol Chem 273: 32230–32235. 9822701 10.1074/jbc.273.48.32230

[16] Collinge J, Sidle KC, Meads J, Ironside J, Hill AF (1996) Molecular analysis of prion strain variation and the aetiology of ‘new variant’ CJD. Nature 383: 685–690. 8878476 10.1038/383685a0

[17] Hill AF, Sidle KC, Joiner S, Keyes P, Martin TC, et al (1998) Molecular screening of sheep for bovine spongiform encephalopathy. Neurosci Lett 255: 159–162. 9832197 10.1016/s0304-3940(98)00736-8

[18] Parchi P, Castellani R, Capellari S, Ghetti B, Young K, et al (1996) Molecular basis of phenotypic variability in sporadic Creutzfeldt-Jakob disease. Ann Neurol 39: 767–778. 8651649 10.1002/ana.410390613

[19] Safar J, Wille H, Itri V, Groth D, Serban H, et al (1998) Eight prion strains have PrP(Sc) molecules with different conformations. Nat Med 4: 1157–1165. 9771749 10.1038/2654

[20] Telling GC, Parchi P, DeArmond SJ, Cortelli P, Montagna P, et al (1996) Evidence for the conformation of the pathologic isoform of the prion protein enciphering and propagating prion diversity. Science 274: 2079–2082. 8953038 10.1126/science.274.5295.2079

[21] Parchi P, Chen SG, Brown P, Zou W, Capellari S, et al (1998) Different patterns of truncated prion protein fragments correlate with distinct phenotypes in P102L Gerstmann-Straussler-Scheinker disease. Proc Natl Acad Sci U S A 95: 8322–8327. 9653185 10.1073/pnas.95.14.8322 PMC20974

[22] Piccardo P, Dlouhy SR, Lievens PM, Young K, Bird TD, et al (1998) Phenotypic variability of Gerstmann-Straussler-Scheinker disease is associated with prion protein heterogeneity. J Neuropathol Exp Neurol 57: 979–988. 9786248 10.1097/00005072-199810000-00010

[23] Piccardo P, Liepnieks JJ, William A, Dlouhy SR, Farlow MR, et al (2001) Prion proteins with different conformations accumulate in Gerstmann-Straussler-Scheinker disease caused by A117V and F198S mutations. Am J Pathol 158: 2201–2207. 11395398 10.1016/S0002-9440(10)64692-5 PMC1891977

[24] Piccardo P, Seiler C, Dlouhy SR, Young K, Farlow MR, et al (1996) Proteinase-K-resistant prion protein isoforms in Gerstmann-Straussler-Scheinker disease (Indiana kindred). J Neuropathol Exp Neurol 55: 1157–1163. 8939199 10.1097/00005072-199611000-00007

[25] Tagliavini F, Lievens PM, Tranchant C, Warter JM, Mohr M, et al (2001) A 7-kDa prion protein (PrP) fragment, an integral component of the PrP region required for infectivity, is the major amyloid protein in Gerstmann-Straussler-Scheinker disease A117V. J Biol Chem 276: 6009–6015. 11087738 10.1074/jbc.M007062200

[26] Tagliavini F, Prelli F, Ghiso J, Bugiani O, Serban D, et al (1991) Amyloid protein of Gerstmann-Straussler-Scheinker disease (Indiana kindred) is an 11 kd fragment of prion protein with an N-terminal glycine at codon 58. EMBO J 10: 513–519. 1672107 10.1002/j.1460-2075.1991.tb07977.x PMC452678

[27] Tagliavini F, Prelli F, Porro M, Rossi G, Giaccone G, et al (1994) Amyloid fibrils in Gerstmann-Straussler-Scheinker disease (Indiana and Swedish kindreds) express only PrP peptides encoded by the mutant allele. Cell 79: 695–703. 7954833 10.1016/0092-8674(94)90554-1

[28] Muramoto T, Tanaka T, Kitamoto N, Sano C, Hayashi Y, et al (2000) Analyses of Gerstmann-Straussler syndrome with 102Leu219Lys using monoclonal antibodies that specifically detect human prion protein with 219Glu. Neurosci Lett 288: 179–182. 10889337 10.1016/s0304-3940(00)01232-5

[29] Wadsworth JD, Joiner S, Linehan JM, Cooper S, Powell C, et al (2006) Phenotypic heterogeneity in inherited prion disease (P102L) is associated with differential propagation of protease-resistant wild-type and mutant prion protein. Brain 129: 1557–1569. 16597650 10.1093/brain/awl076

[30] Monaco S, Fiorini M, Farinazzo A, Ferrari S, Gelati M, et al (2012) Allelic origin of protease-sensitive and protease-resistant prion protein isoforms in Gerstmann-Straussler-Scheinker disease with the P102L mutation. PLoS One 7: e32382. 22384235 10.1371/journal.pone.0032382 PMC3285667

[31] Gambetti P, Dong Z, Yuan J, Xiao X, Zheng M, et al (2008) A novel human disease with abnormal prion protein sensitive to protease. Ann Neurol 63: 697–708. 18571782 10.1002/ana.21420 PMC2767200

[32] Zou WQ, Puoti G, Xiao X, Yuan J, Qing L, et al (2010) Variably protease-sensitive prionopathy: a new sporadic disease of the prion protein. Ann Neurol 68: 162–172. 20695009 10.1002/ana.22094 PMC3032610

[33] Gambetti P, Puoti G, Zou WQ (2011) Variably protease-sensitive prionopathy: a novel disease of the prion protein. J Mol Neurosci 45: 422–424. 21584652 10.1007/s12031-011-9543-1

[34] Xiao X, Yuan J, Haïk S, Cali I, Zhan Y, et al (2013) Glycoform-selective prion formation in sporadic and familial forms of prion disease. PLoS One 8: e58786. 23527023 10.1371/journal.pone.0058786 PMC3602448

[35] Benestad SL, Arsac JN, Goldmann W, Noremark M (2008) Atypical/Nor98 scrapie: properties of the agent, genetics, and epidemiology. Vet Res 39: 19. 18187032 10.1051/vetres:2007056

[36] Benestad SL, Sarradin P, Thu B, Schonheit J, Tranulis MA, et al (2003) Cases of scrapie with unusual features in Norway and designation of a new type, Nor98. Vet Rec 153: 202–208. 12956297 10.1136/vr.153.7.202

[37] Bruce ME, Nonno R, Foster J, Goldmann W, Di Bari M, et al (2007) Nor98-like sheep scrapie in the United Kingdom in 1989. Vet Rec 160: 665–666. 17496276 10.1136/vr.160.19.665

[38] Mitchell GB, O’Rourke KI, Harrington NP, Soutyrine A, Simmons MM, et al (2010) Identification of atypical scrapie in Canadian sheep. J Vet Diagn Invest 22: 408–411. 20453215 10.1177/104063871002200310

[39] Loiacono CM, Thomsen BV, Hall SM, Kiupel M, Sutton D, et al (2009) Nor98 scrapie identified in the United States. J Vet Diagn Invest 21: 454–463. 19564493 10.1177/104063870902100406

[40] Kittelberger R, Chaplin MJ, Simmons MM, Ramirez-Villaescusa A, McIntyre L, et al (2010) Atypical scrapie/Nor98 in a sheep from New Zealand. J Vet Diagn Invest 22: 863–875. 21088169 10.1177/104063871002200604

[41] Fediaevsky A, Tongue SC, Noremark M, Calavas D, Ru G, et al (2008) A descriptive study of the prevalence of atypical and classical scrapie in sheep in 20 European countries. BMC Vet Res 4: 19. 18544152 10.1186/1746-6148-4-19 PMC2442063

[42] McIntyre KM, del Rio Vilas VJ, Gubbins S (2008) No temporal trends in the prevalence of atypical scrapie in British sheep, 2002–2006. BMC Vet Res 4: 13. 18384678 10.1186/1746-6148-4-13 PMC2397389

[43] Colussi S, Vaccari G, Maurella C, Bona C, Lorenzetti R, et al (2008) Histidine at codon 154 of the prion protein gene is a risk factor for Nor98 scrapie in goats. J Gen Virol 89: 3173–3176. 19008408 10.1099/vir.0.2008/004150-0

[44] Moum T, Olsaker I, Hopp P, Moldal T, Valheim M, et al (2005) Polymorphisms at codons 141 and 154 in the ovine prion protein gene are associated with scrapie Nor98 cases. J Gen Virol 86: 231–235. 15604451 10.1099/vir.0.80437-0

[45] Fediaevsky A, Maurella C, Noremark M, Ingravalle F, Thorgeirsdottir S, et al (2010) The prevalence of atypical scrapie in sheep from positive flocks is not higher than in the general sheep population in 11 European countries. BMC Vet Res 6: 9. 20137097 10.1186/1746-6148-6-9 PMC2832631

[46] European Commission D-GfHaC, Directorate G-Veterinary and International Affairs, Unit G4-Food, Alert System and Training (2011) Report on the monitoring and testing of ruminants for the presence of Transmissible Spongiform Encephalopathy (TSE) in the EU in 2010.

[47] Le Dur A, Beringue V, Andreoletti O, Reine F, Lai TL, et al (2005) A newly identified type of scrapie agent can naturally infect sheep with resistant PrP genotypes. Proc Natl Acad Sci U S A 102: 16031–16036. 16239348 10.1073/pnas.0502296102 PMC1276041

[48] Simmons MM, Konold T, Simmons HA, Spencer YI, Lockey R, et al (2007) Experimental transmission of atypical scrapie to sheep. BMC Vet Res 3: 20. 17725818 10.1186/1746-6148-3-20 PMC2025597

[49] Simmons MM, Moore SJ, Konold T, Thurston L, Terry LA, et al (2011) Experimental oral transmission of atypical scrapie to sheep. Emerg Infect Dis 17: 848–854. 21529394 10.3201/eid1705.101654 PMC3321785

[50] Andreoletti O, Orge L, Benestad SL, Beringue V, Litaise C, et al (2011) Atypical/Nor98 scrapie infectivity in sheep peripheral tissues. PLoS Pathog 7: e1001285. 21347349 10.1371/journal.ppat.1001285 PMC3037359

[51] Arsac JN, Andreoletti O, Bilheude JM, Lacroux C, Benestad SL, et al (2007) Similar biochemical signatures and prion protein genotypes in atypical scrapie and Nor98 cases, France and Norway. Emerg Infect Dis 13: 58–65. 17370516 10.3201/eid1301.060393 PMC2725815

[52] Everest SJ, Thorne L, Barnicle DA, Edwards JC, Elliott H, et al (2006) Atypical prion protein in sheep brain collected during the British scrapie-surveillance programme. J Gen Virol 87: 471–477. 16432036 10.1099/vir.0.81539-0

[53] Gotte DR, Benestad SL, Laude H, Zurbriggen A, Oevermann A, et al (2011) Atypical scrapie isolates involve a uniform prion species with a complex molecular signature. PLoS One 6: e27510. 22096587 10.1371/journal.pone.0027510 PMC3214077

[54] Gretzschel A, Buschmann A, Langeveld J, Groschup MH (2006) Immunological characterization of abnormal prion protein from atypical scrapie cases in sheep using a panel of monoclonal antibodies. J Gen Virol 87: 3715–3722. 17098989 10.1099/vir.0.81816-0

[55] Klingeborn M, Wik L, Simonsson M, Renstrom LH, Ottinger T, et al (2006) Characterization of proteinase K-resistant N- and C-terminally truncated PrP in Nor98 atypical scrapie. J Gen Virol 87: 1751–1760. 16690942 10.1099/vir.0.81618-0

[56] Mazza M, Iulini B, Vaccari G, Acutis PL, Martucci F, et al (2010) Co-existence of classical scrapie and Nor98 in a sheep from an Italian outbreak. Res Vet Sci 88: 478–485. 20031179 10.1016/j.rvsc.2009.11.015

[57] Wemheuer WM, Benestad SL, Wrede A, Schulze-Sturm U, Wemheuer WE, et al (2009) Similarities between forms of sheep scrapie and Creutzfeldt-Jakob disease are encoded by distinct prion types. Am J Pathol 175: 2566–2573. 19850886 10.2353/ajpath.2009.090623 PMC2789619

[58] Pirisinu L, Di Bari M, Marcon S, Vaccari G, D’Agostino C, et al (2010) A new method for the characterization of strain-specific conformational stability of protease-sensitive and protease-resistant PrP. PLoS One 5: e12723. 20856860 10.1371/journal.pone.0012723 PMC2939050

[59] Migliore S, Esposito E, Pirisinu L, Marcon S, Di Bari M, et al (2012) Effect of PrP genotype and route of inoculation on the ability of discriminatory Western blot to distinguish scrapie from sheep bovine spongiform encephalopathy. J Gen Virol 93: 450–455. 21994325 10.1099/vir.0.035469-0

[60] Pirisinu L, Migliore S, Di Bari MA, Esposito E, Baron T, et al (2011) Molecular discrimination of sheep bovine spongiform encephalopathy from scrapie. Emerg Infect Dis 17: 695–698. 21470463 10.3201/eid1704.101215 PMC3377410

[61] Feraudet C, Morel N, Simon S, Volland H, Frobert Y, et al (2005) Screening of 145 anti-PrP monoclonal antibodies for their capacity to inhibit PrPSc replication in infected cells. J Biol Chem 280: 11247–11258. 15618225 10.1074/jbc.M407006200

[62] Jeffrey M, Martin S, Gonzalez L, Foster J, Langeveld JP, et al (2006) Immunohistochemical features of PrP(d) accumulation in natural and experimental goat transmissible spongiform encephalopathies. J Comp Pathol 134: 171–181. 16542672 10.1016/j.jcpa.2005.10.003

[63] Yull HM, Ritchie DL, Langeveld JP, van Zijderveld FG, Bruce ME, et al (2006) Detection of type 1 prion protein in variant Creutzfeldt-Jakob disease. Am J Pathol 168: 151–157. 16400018 10.2353/ajpath.2006.050766 PMC1592651

[64] Miller MB, Geoghegan JC, Supattapone S (2011) Dissociation of infectivity from seeding ability in prions with alternate docking mechanism. PLoS Pathog 7: e1002128. 21779169 10.1371/journal.ppat.1002128 PMC3136465

[65] Pankiewicz J, Prelli F, Sy MS, Kascsak RJ, Kascsak RB, et al (2006) Clearance and prevention of prion infection in cell culture by anti-PrP antibodies. Eur J Neurosci 23: 2635–2647. 16817866 10.1111/j.1460-9568.2006.04805.x PMC1779824

[66] O’Rourke KI, Baszler TV, Miller JM, Spraker TR, Sadler-Riggleman I, et al (1998) Monoclonal antibody F89/160.1.5 defines a conserved epitope on the ruminant prion protein. J Clin Microbiol 36: 1750–1755. 9620413 10.1128/jcm.36.6.1750-1755.1998 PMC104913

[67] Parchi P, Zou W, Wang W, Brown P, Capellari S, et al (2000) Genetic influence on the structural variations of the abnormal prion protein. Proc Natl Acad Sci U S A 97: 10168–10172. 10963679 10.1073/pnas.97.18.10168 PMC27779

[68] Polymenidou M, Prokop S, Jung HH, Hewer E, Peretz D, et al (2011) Atypical prion protein conformation in familial prion disease with PRNP P105T mutation. Brain Pathol 21: 209–214. 20875062 10.1111/j.1750-3639.2010.00439.x PMC8094255

[69] Choi YP, Peden AH, Groner A, Ironside JW, Head MW (2010) Distinct stability states of disease-associated human prion protein identified by conformation-dependent immunoassay. J Virol 84: 12030–12038. 20844046 10.1128/JVI.01057-10 PMC2977900

[70] Vulin J, Biacabe AG, Cazeau G, Calavas D, Baron T (2011) Molecular typing of protease-resistant prion protein in transmissible spongiform encephalopathies of small ruminants, France, 2002–2009. Emerg Infect Dis 17: 55–63. 21192855 10.3201/eid1701.100891 PMC3204636

[71] Jacobs JG, Langeveld JP, Biacabe AG, Acutis PL, Polak MP, et al (2007) Molecular discrimination of atypical bovine spongiform encephalopathy strains from a geographical region spanning a wide area in Europe. J Clin Microbiol 45: 1821–1829. 17442800 10.1128/JCM.00160-07 PMC1933055

[72] Cali I, Castellani R, Alshekhlee A, Cohen Y, Blevins J, et al (2009) Co-existence of scrapie prion protein types 1 and 2 in sporadic Creutzfeldt-Jakob disease: its effect on the phenotype and prion-type characteristics. Brain 132: 2643–2658. 19734292 10.1093/brain/awp196 PMC2766234

[73] Kim C, Haldiman T, Cohen Y, Chen W, Blevins J, et al (2011) Protease-sensitive conformers in broad spectrum of distinct PrPSc structures in sporadic Creutzfeldt-Jakob disease are indicator of progression rate. PLoS Pathog 7: e1002242. 21931554 10.1371/journal.ppat.1002242 PMC3169556

[74] Gielbert A, Davis LA, Sayers AR, Hope J, Gill AC, et al (2009) High-resolution differentiation of transmissible spongiform encephalopathy strains by quantitative N-terminal amino acid profiling (N-TAAP) of PK-digested abnormal prion protein. J Mass Spectrom 44: 384–396. 19053160 10.1002/jms.1516

[75] Tang Y, Gielbert A, Jacobs JG, Baron T, Andreoletti O, et al (2012) All major prion types recognised by a multiplex immunofluorometric assay for disease screening and confirmation in sheep. J Immunol Methods 380: 30–39. 22498749 10.1016/j.jim.2012.03.004

[76] Griffiths PC, Spiropoulos J, Lockey R, Tout AC, Jayasena D, et al (2010) Characterization of atypical scrapie cases from Great Britain in transgenic ovine PrP mice. J Gen Virol 91: 2132–2138. 20392900 10.1099/vir.0.018986-0

[77] Piccardo P, Manson JC, King D, Ghetti B, Barron RM (2007) Accumulation of prion protein in the brain that is not associated with transmissible disease. Proc Natl Acad Sci U S A 104: 4712–4717. 17360589 10.1073/pnas.0609241104 PMC1838665

[78] Bruce ME, Will RG, Ironside JW, McConnell I, Drummond D, et al (1997) Transmissions to mice indicate that ‘new variant’ CJD is caused by the BSE agent. Nature 389: 498–501. 9333239 10.1038/39057

[79] Tranulis MA, Benestad SL, Baron T, Kretzschmar H (2011) Atypical prion diseases in humans and animals. Top Curr Chem 305: 23–50. 21598097 10.1007/128_2011_161

[80] Baker HF, Duchen LW, Jacobs JM, Ridley RM (1990) Spongiform encephalopathy transmitted experimentally from Creutzfeldt-Jakob and familial Gerstmann-Straussler-Scheinker diseases. Brain 113 (Pt 6): 1891–1909. 10.1093/brain/113.6.1891 2276050

[81] Tateishi J, Kitamoto T, Hoque MZ, Furukawa H (1996) Experimental transmission of Creutzfeldt-Jakob disease and related diseases to rodents. Neurology 46: 532–537. 8614527 10.1212/wnl.46.2.532

[82] Arsac JN, Betemps D, Morignat E, Feraudet C, Bencsik A, et al (2009) Transmissibility of atypical scrapie in ovine transgenic mice: major effects of host prion protein expression and donor prion genotype. PLoS One 4: e7300. 19806224 10.1371/journal.pone.0007300 PMC2752806

[83] Espinosa JC, Herva ME, Andreoletti O, Padilla D, Lacroux C, et al (2009) Transgenic mice expressing porcine prion protein resistant to classical scrapie but susceptible to sheep bovine spongiform encephalopathy and atypical scrapie. Emerg Infect Dis 15: 1214–1221. 19751582 10.3201/eid1508.081218 PMC2815954

[84] Yuan J, Dong Z, Guo JP, McGeehan J, Xiao X, et al (2008) Accessibility of a critical prion protein region involved in strain recognition and its implications for the early detection of prions. Cell Mol Life Sci 65: 631–643. 18193391 10.1007/s00018-007-7478-z PMC7079802

[85] Zou WQ, Langeveld J, Xiao X, Chen S, McGeer PL, et al (2010) PrP conformational transitions alter species preference of a PrP-specific antibody. J Biol Chem 285: 13874–13884. 20194495 10.1074/jbc.M109.088831 PMC2859550

