# Supplementary figures and images for: Small Ruminant Nor98 Prions Share Biochemical Features with Human Gerstmann-Sträussler-Scheinker Disease and Variably Protease-Sensitive Prionopathy
Source: PLoS One. 2013 Jun 24;8(6):e66405. doi: 10.1371/journal.pone.0066405 (PMC3691246; doi:10.1371/journal.pone.0066405)

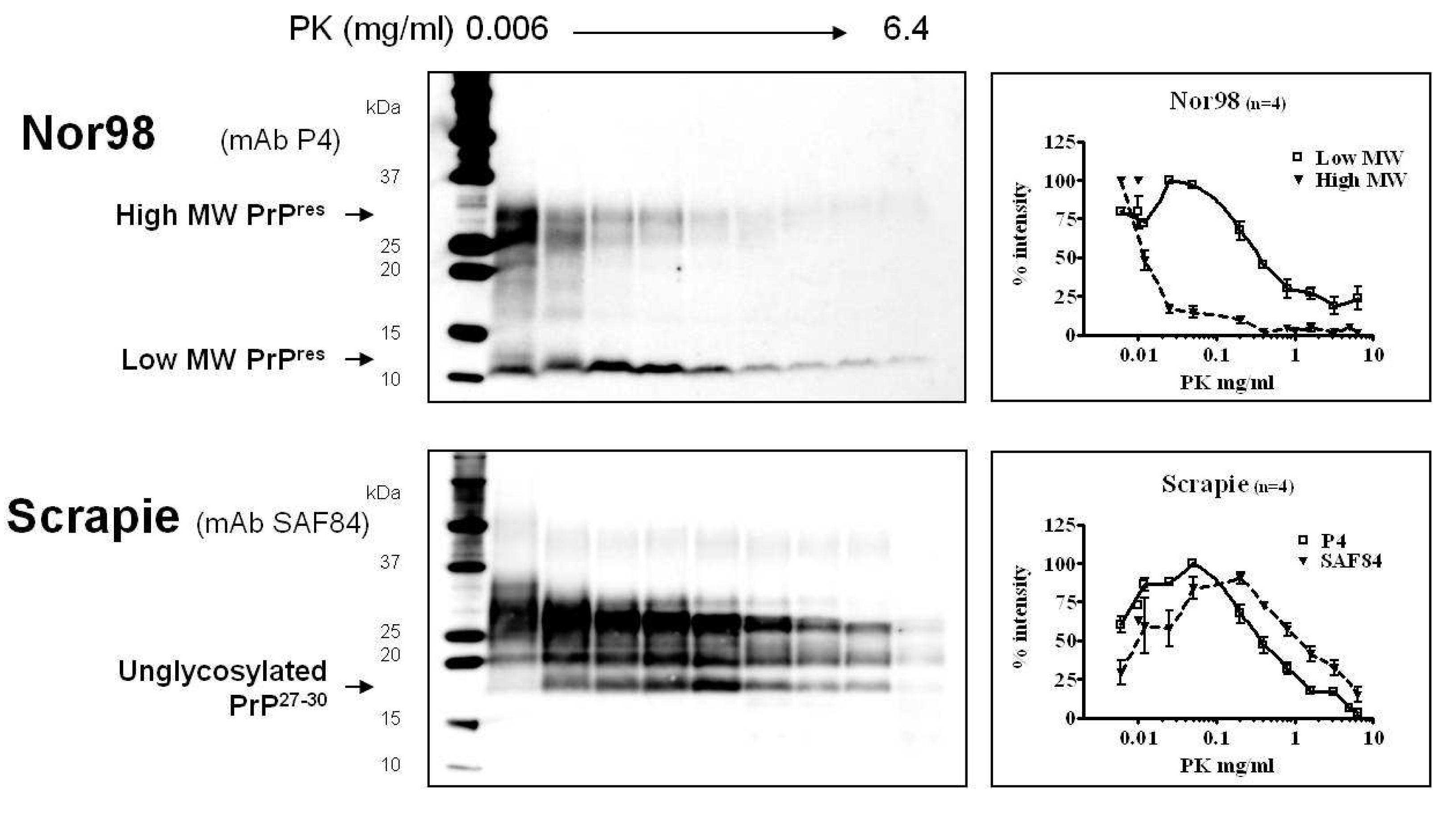

Supplement: Figure S1 — Characterization of Nor98 PrPres fragments. Comparative PK titration in Nor98 and classical scrapie. PK digestion curves of classical scrapie (n = 4) and Nor98 (n = 4) with concentrations of PK ranging from 0.006 to 6.4 mg/ml. Replica blots were probed with SAF84 and P4 and selected fragments were quantified. Representative WB are shown in the left panels, and the quantitative analysis of PrPres for the determination of the PK1/2 on the right panels. For quantitative purposes, both high and low MW PrPres were measured in Nor98, while the unglycosylated PrP27–30 was measured in scrapie. In Nor98 (upper panels), there was a clear-cut distinction between fragments with different resistance to proteolysis. High MW fragments displayed very low PK1/2, ranging from 0.01 to 0.03 mg/ml, while the P4-positive low MW fragments were still detected at the highest PK-concentrations. Low MW PrPres amount increased up to 0.025 mg/ml PK and then declined, similarly to what observed with PrP27–30 in classical scrapie. The PK1/2 of low MW PrPres ranged from 0.21 to 0.53 mg/ml PK. In classical scrapie (lower panels), the levels of PrP27–30 reached a plateau between 0.012 and 0.05 mg/ml PK with P4 and between 0.012 and 0.2 mg/ml PK with SAF84; afterwards the quantity of PrP27–30 declined with both mAbs. Interestingly, the decline of SAF84-positive PrPres was parallel but shifted to the right compared to that measured by P4. Quantitative analysis showed that the PK1/2 for degradation of PrP27–30 ranged from 0.24 to 0.68 mg/ml with P4 and from 1 to 2.1 mg/ml with SAF84. (TIF) [file pone.0066405.s001.tif]

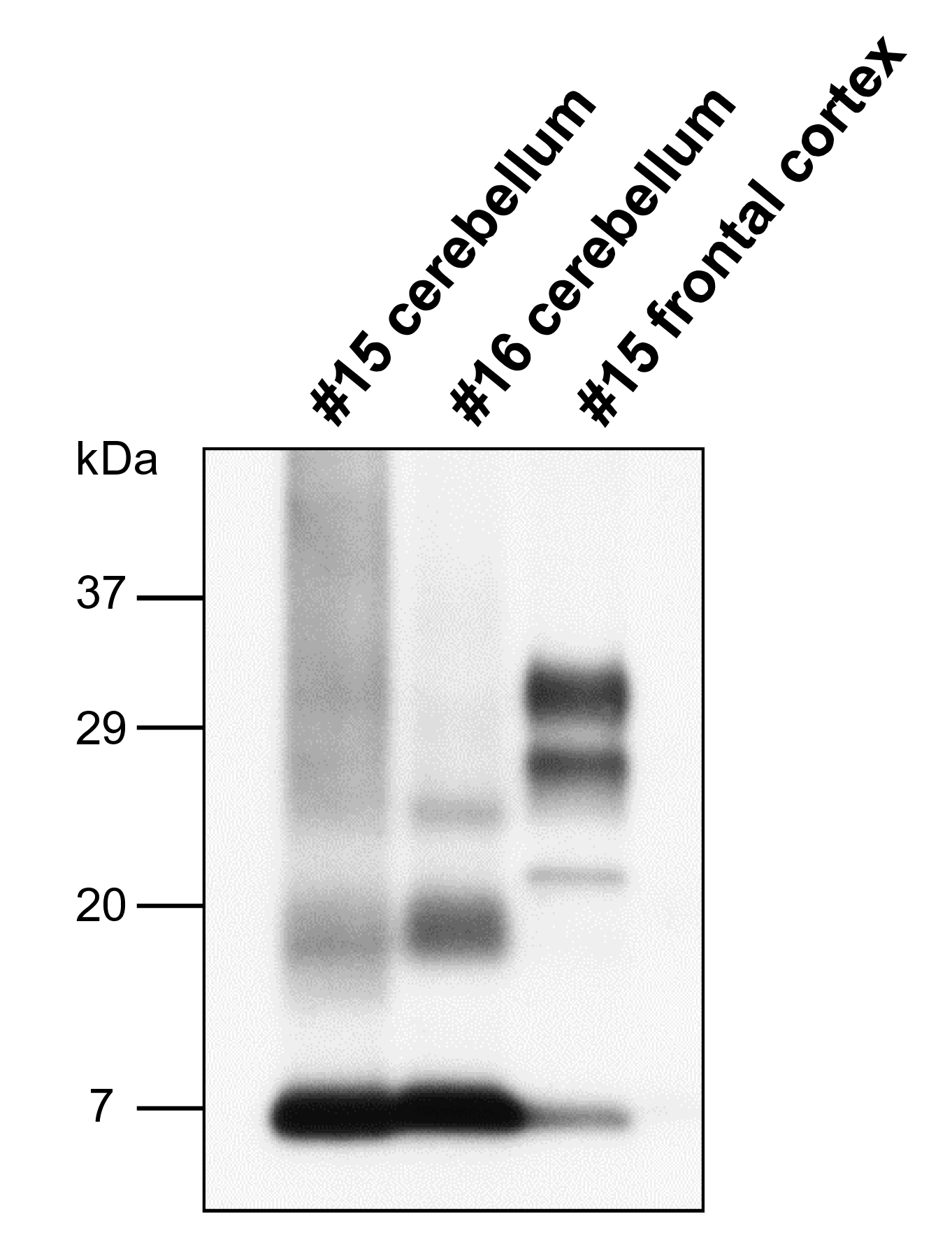

Supplement: Figure S2 — PrPres phenotypes in GSS P102L cases. Western blot of the two GSS P102L cases (#15 and #16, Table 1). Samples were treated with 50 µg/ml PK and membranes were probed with F89. MW markers are shown in kilodaltons on the left. (TIF) [file pone.0066405.s002.tif]

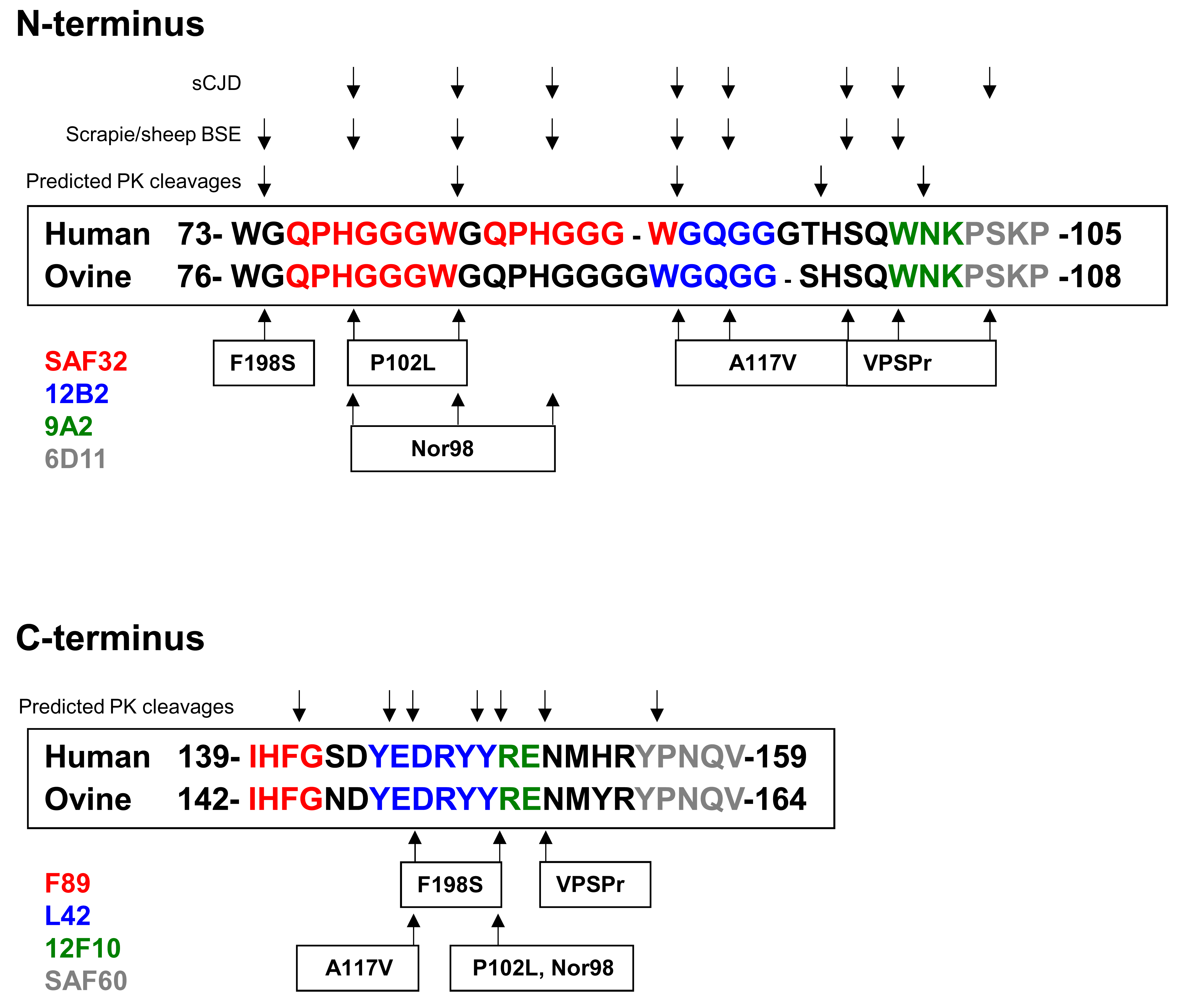

Supplement: Figure S3 — Derivation of the N and C terminal PK cleavage sites from epitope mapping data. The PK cleavages were derived taking into account the epitope mapping data, summarised in Table 2, the known N-terminal cleavage sites in sCJD [67] and in scrapie or sheep BSE [74], [75], and the potential cleavage sites cleaved by PK in the corresponding human and sheep PrP sequences, as predicted by the PeptideCutter software (ExPASy). The cleavage sites used for our determination are represented by arrows on the top of the human and sheep aa sequences, while the derived cleavage sites for GSS, VPSPr and Nor98 are represented by arrows below the aa sequences. Coloured letters highlight the epitopes of the relevant mAbs (reported in the figure with the corresponding colour) used in epitope mapping experiments. When epitopes of mAbs partially overlap, only the aa differentiating the epitopes were coloured (for a full description of epitopes see Table 2). For the sake of clarity, in all instances where two consecutive amino acids where deemed as possible cleavage sites, only one of them was reported in the figure. At the N-terminus (upper panel), VPSPr PrPres did not included SAF32 and 12B2 epitopes and only partially included the 9A2 epitope, and thus the derived cleavage sites were those before and after the 9A2 epitope, i.e. S97, W99 and S103, corresponding to cleavage sites detected in type 2 sCJD. GSS A117V PrPres included the 9A2 epitope, but not completely the 12B2, with derived cleavage sites G90, G92 and S97, corresponding to cleavage sites detected in type MV2 and VV2 sCJD. For the other PrPres types having 12B2 and SAF32 epitopes it was less obvious to derive potential cleavage sites. Indeed, SAF32 recognises an epitope repeated 4 times within the OR sequence, so that for SAF32-positive samples could have been cleaved at several positions within the OR sequence. However, based on the N-terminal sequencing reported for sCJD and scrapie and on the apparent MW observed in WB, we c [file pone.0066405.s003.tif]
