# Supplementary material for: Small Ruminant Nor98 Prions Share Biochemical Features with Human Gerstmann-Sträussler-Scheinker Disease and Variably Protease-Sensitive Prionopathy
Source: PLoS One. 2013 Jun 24;8(6):e66405. doi: 10.1371/journal.pone.0066405 (PMC3691246; doi:10.1371/journal.pone.0066405)
Supplement: Table S1 — Table with sheep and goat samples. (DOC) [file pone.0066405.s004.doc]

**Table S1. Table with sheep and goat samples.**

| **Strain** | **Origin** | **Species (n)** | **PrP genotype* (n)** |
| --- | --- | --- | --- |
|  |  |  |  |
| *Nor98* | Italy | sheep (12) | ALRQ/ALHQ (2) |
|  |  |  | ALRQ/AFRQ (2) |
|  |  |  | AFRQ/AFRQ (2) |
|  |  |  | ALRR/AFRQ (2) |
|  |  |  | ALRR/ALHQ (2) |
|  |  |  | ALRR/ALRR (2) |
|  |  | goat (4) | ALRQ/ALHQ (2) |
|  |  |  | ALHQ/ALHQ (2) |
|  |  |  |  |
|  | Norway | sheep (10) | ALRQ/ALHQ (1) |
|  |  |  | AFRQ/AFRQ (3) |
|  |  |  | AFRQ/ALHQ (2) |
|  |  |  | ALHQ/ALHQ (4) |
|  |  |  |  |
|  |  |  |  |
| *Classical Scrapie* | Italy | sheep (6) | ALRQ/ALRQ (2) |
|  |  |  | ALRQ/ALHQ (2) |
|  |  |  | ALRQ/AFRQ (2) |
|  |  | goat (4) | ALRQ/ALRQ (4) |
|  |  |  |  |

*PrP genotype at sheep polymorphic codons 136, 141, 154 and 171. The same nomenclature was also used for goat PrP, although aa positions 136, 141 and 171 are not polymorphic in this species.
